# Time series clustering of T cell subsets dissects heterogeneity in immune reconstitution and clinical outcomes among MUD-HCT patients receiving ATG or PTCy

**DOI:** 10.3389/fimmu.2023.1082727

**Published:** 2023-03-20

**Authors:** Saskia Leserer, Theresa Graf, Martina Franke, Rashit Bogdanov, Esteban Arrieta-Bolaños, Ulrike Buttkereit, Nils Leimkühler, Katharina Fleischhauer, Hans Christian Reinhardt, Dietrich W. Beelen, Amin T. Turki

**Affiliations:** ^1^ Department of Hematology and Stem Cell Transplantation, West-German Cancer Center, University Hospital Essen, Essen, Germany; ^2^ Computational Hematology Lab, Department of Hematology and Stem Cell Transplantation, West-German Cancer Center, University Hospital Essen, Essen, Germany; ^3^ Institute for Experimental Cellular Therapy, West-German Cancer Center, University Hospital Essen, Essen, Germany; ^4^ German Cancer Consortium Deutsches Konsortium für Translationale Krebsforschung (DKTK), Partner site Essen/Düsseldorf, Essen, Germany; ^5^ Cancer Research Center Cologne Essen (CCCE), Essen, Germany

**Keywords:** GVHD prophylaxis, anti-thymocyte globulin (ATG), post-transplant cyclophosphamide, unsupervised learning, matched unrelated donor allogeneic hematopoietic stem cell transplantation, anti-T-lymphocyte globulin, time-series (TS) model, dynamic time warping (DTW)

## Abstract

**Introduction:**

Anti-T-lymphocyte globulin (ATG) or post-transplant cyclophosphamide (PTCy) prevent graft-versus-host disease (GVHD) after hematopoietic cell transplantation (HCT), yet individual patients benefit differentially.

**Methods:**

Given the sparse comparative data on the impact of cellular immune reconstitution in this setting, we studied flow cytometry and clinical outcomes in 339 recipients of 10/10 matched-unrelated donor (MUD) HCT using either ATG (n=304) or PTCy (n=35) for *in vivo* T cell manipulation along with a haploidentical PTCy control cohort (n=45). Longitudinal cellular immune reconstitution data were analyzed conventionally and with a data science approach using clustering with dynamic time warping to determine the similarity between time-series of T cell subsets.

**Results:**

Consistent with published studies, no significant differences in clinical outcomes were observed at the cohort level between MUD-ATG and MUD-PTCy. However, cellular reconstitution revealed preferences for distinct T cell subpopulations associating with GVHD protection in each setting. Starting early after HCT, MUD-PTCy patients had higher regulatory T cell levels after HCT (p <0.0001), while MUD-ATG patients presented with higher levels of γδ T- or NKT cells (both p <0.0001). Time-series clustering further dissected the patient population’s heterogeneity revealing distinct immune reconstitution clusters. Importantly, it identified phenotypes that reproducibly associated with impaired clinical outcomes within the same *in vivo* T cell manipulation platform. Exemplarily, patients with lower activated- and αβ T cell counts had significantly higher NRM (p=0.032) and relapse rates (p =0.01).

**Discussion:**

The improved understanding of the heterogeneity of cellular reconstitution in MUD patients with T cell manipulation both at the cohort and individual level may support clinicians in managing HCT complications.

## Highlights

1. GVHD prophylaxis with ATG or PTCy leads to preferential expansion of distinct T cell subsets, regulatory T cells or γδ T- and NKT cells
2. Time-series clustering of T cell subsets identifies phenotypes that associated with distinct clinical outcomes within each *in vivo* T cell manipulation platform

## Introduction

Despite the introduction of high-resolution human leukocyte antigen typing for donor selection, graft-versus-host disease (GVHD) remains one of the most frequent complications and a major cause of mortality after allogeneic hematopoietic cell transplantation (HCT) (1, 2). The continuous increase of HCT from alternative donor sources, such as matched unrelated donor (MUD), mismatched unrelated donor (MMUD) or haploidentical donors (3) required improved GVHD prophylaxis strategies beyond the use of baseline calcineurin inhibitors and antimetabolites. Proliferating alloreactive T cells are considered to be the leading mediators of acute GVHD (aGVHD) (4), to contribute to the pathogenesis of chronic GVHD (cGVHD) (5) and are hence promising targets for preventing excessive alloreactivity. During the last decade, the addition of *in vivo* T cell depletion using anti-T-lymphocyte globulin (ATG) or alemtuzumab have become the standard-of-care in MUD-HCT in most European centers (6). More recently, post-transplant cyclophosphamide (PTCy) has proven to be a safe and feasible alternative for GVHD prophylaxis in patients with haploidentical- (7–9), MUD- (10, 11), or MMUD donors (12, 13). Hence, previous studies have compared the efficacy of ATG and PTCy as GVHD prophylaxis in different HCT settings, showing comparable GVHD incidences in haploidentical patients (14) and even lower incidences of aGVHD II-IV in unrelated donor-HCT with PTCy (15). While the clinical impact of both agents has been well scrutinized, comparative immune reconstitution studies are scarce and provided differential results in cohorts with distinct conditioning (16, 17) or donor settings. ATG is well known to delay the reconstitution of CD3^+^ and CD4^+^ T cells, in particular of T helper cells, up to 12 months post-HCT (18, 19), while PTCy preserves regulatory T cells (Tregs) thus allowing their rapid recovery (20). A sufficient reconstitution of CD4^+^ T cells after HCT previously associated with lower mortality (21). Similarly, early helper T cell reconstitution and clinical patient outcome were improved by optimized dosing of ATG (19, 22). Improved understanding of differential effects of ATG or PTCy on heterogeneous cellular immune reconstitution might support HCT physicians in managing GVHD prophylaxis in different donor settings. Based on this hypothesis we compared patients with MUD HCT using either ATG or PTCy as GVHD prophylaxis. As the PTCy platform has been originally developed for haploidentical HCT (23), we added a control cohort with haplo-PTCy. Beyond cohort comparisons, we leveraged time series clustering on longitudinal T cell reconstitution data with the purpose to dissect the interindividual heterogeneity in immune reconstitution and to better differentiate clinical outcomes in patients receiving the same GVHD prophylaxis.

## Methods

### Study population

The study population was selected from 551 consecutive patients with allogeneic HCT between January 2017 and May 2020 at the Department of Hematology and Stem Cell Transplantation of the West-German Cancer Center, University Hospital Essen. Patients were screened for the following inclusion criteria: administration of *in vivo* T cell manipulation with 1) ATG or 2) PTCy as GVHD prophylaxis for HCT from 10/10 matched-unrelated (MUD) donors (CONSORT diagram, **Supplementary Figure 1A**). All patients received peripheral blood stem cells (PBSC) as graft. Given that graft failure biases the analysis of donor-derived immune reconstitution, 9 patients with graft failure were excluded prior analysis. Patients transplanted with haploidentical donors treated with PTCy were included as comparators. A total of 384 patients were eligible for downstream analysis.

GVHD prophylaxis consisted of baseline calcineurin inhibitor-based immunosuppression combined with *in vivo* T cell manipulation using either ATG or PTCy. ATG (Grafalon®, Neovii, Rapperswill, CH) (n=304) was applied at a dose of 10mg/kg or 20mg/kg bodyweight on three consecutive days between day -4 and day -2 before HCT based on standardized protocols, followed by ciclosporin and methotrexate starting at day -1. PTCy (n=80) was administered on day +3 and +4 (50 mg/kg body weight per day) post-HCT followed by tacrolimus and mycophenolate-mofetil (MMF) starting on day +5. Out of these 80 patients receiving PTCy as GVHD prophylaxis 35 patients (44%) were transplanted with MUD donors and 45 patients (56%) with haploidentical donors. Early supportive and follow-up care followed the same internal protocols and was considered identical for all patients. Patients were followed-up until the last documented clinical assessment or death by any cause. Surviving patients were censored at maximum follow-up of 12 months. The clinical assessment standards are detailed in the **Supplementary Methods**.

### Patient assessment

Baseline data concerning patient-, donor-, allogeneic hematopoietic cell transplantation (HCT) characteristics and HCT-outcome were documented prospectively in electronic forms. Laboratory results and clinical characteristics of patients after HCT were retrospectively analyzed. Clinical assessment was obtained daily for inpatients and at each outpatient visit, starting with weekly intervals. Acute GVHD (aGVHD) was defined as GVHD organ involvement of skin, gut and/or liver until 100 days post-HCT. aGVHD was clinically assessed and classified according to the consensus aGVHD (24) grading. Diagnosis of chronic GVHD (cGVHD) starting from day +100 was based on characteristic symptoms and clinical signs according to the published NIH criteria (25). Overall survival was defined as the time from HCT to the end of the 12-months follow-up period or up to death by any cause. Cumulative incidence of relapse incidence (CIR) was calculated as the time from the day of transplantation to the day of documented relapse to original disease or persistence of malignancy. For patients without relapse or persisting malignancy, non-relapse mortality (NRM) was determined as the time from day of HCT to death.

### Monitoring of immune reconstitution, comparative analysis, and time-series clustering

Immune reconstitution after MUD HCT was studied in peripheral blood samples from patients around months +1, +3, +6, +9, and +12 after HCT. A total of 1297 samples were analyzed by flow cytometry at the BMT Laboratory, University Hospital Essen. For flow cytometry analysis, freshly-derived patient peripheral blood samples were prepared by isolating mononuclear cells (PBMC) using an automated red blood cell lysing system (TQ-Prep, Beckman Coulter, Brea, CA), washing with fluorescence-activated cell sorting (FACS) buffer and subsequently staining with surface markers (**Supplementary Table 1**). No samples were cryopreserved. All samples were run on the same NAVIOS flow cytometer (Beckman Coulter, Brea, CA) using the same antibodies and FACS compensation parameters using the manufacturer’s software. Adequate subset representation was ensured by analysis of a minimum of 15000 lymphocytes in each run. For each flow cytometry sample two complementary antibody panels were used. The first panel characterized immune cell subsets as follows: T Cells, CD3^+^; T helper cells, CD3^+^/CD4^+^; cytotoxic T cells, CD3^+^/CD8^+^; regulatory T cells, CD3^+^/CD4^+^/CD25^+^/CD127^+^low; conventional T cells, CD3^+^/CD4^+^/CD25^-^/CD127^+^high, not including the CD3^+^/CD4^+^/CD25^-^/CD127^-^low fraction; naïve helper T cells, CD3^+^/CD4^+^/CD45RA^+^; memory helper T cells, CD3^+^/CD4^+^/CD45RO^+^. Given that the panel did not cover CCR7 or CD62L, we described the CD8**^+^** population including both naïve cytotoxic T cells and effector memory T cells re-expressing CD45RA (TEMRA) as CD45RA^+^ cytotoxic T cells, CD3^+^/CD8^+^/CD45RA^+^. Memory cytotoxic T cells were characterized by CD3^+^/CD8^+^/CD45RO^+^ and B cells by CD19^+^. These subsets were gated on the CD45^+^ lymphocyte gate, excepting the regulatory- and conventional T cells, which were selected from the CD3^+^/CD4^+^ subset in the CD45+ gate. In the second panel the following immune cell subsets were gated on the CD45^+^ lymphocyte gate: Activated T cells, CD3^+^/HLA-DR^+^; NKG2D^+^-NK cells, CD16^+^/CD56^+^/CD314^+^. T cell receptor α/β, TCRα/β and T cell receptor γ/δ, TCRγ/δ were gated on the CD3^+^ gate. T cell subset counts were determined as fraction of the absolute lymphocyte count on the sampling date. For each individual point in time (**Supplementary Figure 1B**), median counts of immune subsets were compared between MUD-ATG, MUD-PTCy using Mann-Whitney U test (GraphPad Prism 9.0.0, GraphPad Software, LLC, San Diego California). Samples from patients with haplo-HCT served as comparative samples to discriminate PTCy specific effects from those specific to the donor setting.

Detailed information about the methods to analyze individual patient’ longitudinal immune reconstitution is provided in the **Supplementary Methods** section. In short, we defined two distinct multi-dimensional immune cell clustering models integrating two different groups of T cell subsets: 1) “GVHD-associated” T cells: CD3^+^/CD4^+^/CD25^+^/CD127^low^ Treg, CD3^+^/HLA-DR^+^ activated T cells, TCRα/β^+^ and TCRγ/δ^+^ T cells based on immunologic evidence of a mechanistic impact in GVHD (26, 27) and 2) “broad spectrum” T cells: CD3^+^/CD4^+^ helper T cells, CD3^+^/CD4^+^/CD45RA^+^ naïve helper T cells, CD3^+^/CD8^+^ cytotoxic T cells, and CD3^+^/CD8^+^/CD45RO^+^ memory cytotoxic T cells which represent relevant subpopulations of the CD4^+^ and CD8^+^ T cell compartment (28, 29). In order to manage the complexity of the defined models both were limited to four T cell subsets. To apply these models, the study population (n=384) was filtered for patients with 1) at least three flow cytometry measurements within +12 months post-HCT and 2) measurement of the first flow cytometry ≤ d+45 post-HCT. Data filtering resulted in a patient subgroup of 180 patients eligible for clustering analysis (n=147 MUD-ATG, n=15 MUD-PTCy, and n=18 haplo-PTCy; for technical reasons, the “broad spectrum” T cell model included 4 more patients in the MUD-ATG cohort (n=151)). Each patients’ T cell subsets time-series underwent linear interpolation between datapoints (**Supplementary equation 1**), calculated from adjacent values to have continuous data. Individual longitudinal immune reconstitution was then studied within each patient cohort by partitional clustering of time-series data (30, 31). Here, partitional clustering was performed using dynamic time warping (DTW) as distance measure (30) with 36 different function-specific configurations tested (**Supplementary Table 2**). The performance of clustering configurations was evaluated by the silhouette coefficient (*Sil*), indicating a separation of clusters between -1 and +1 with the optimum at +1 (32). Model robustness was internally validated *via* a 10-fold resampling approach (see **Supplementary method** section) examining the variance of the *Sil* for each configuration. Data interpolation, DTW and time-series clustering were performed using R (33) packages *R stats (* 33) and *dtwclust (* 34) (R version 3.6.3, R core Team, https://www.r-project.org/). Patient clusters identified using this approach were evaluated for clinical outcomes as explained in the “Clinical statistical analysis” section.

### Clinical statistical analysis

Patient baseline characteristics were analyzed with Chi-square test and one-way ANOVA where appropriate (GraphPad Prism 9.0.0). The primary clinical outcome of this study was the incidence of grades II-IV aGVHD. Secondary endpoints were the overall incidence of 100-day aGVHD, 1-year relapse and NRM, 1-year cGVHD, as well as 1-year overall survival. The cumulative incidence of all-grade aGVHD and aGVHD II-IV was analyzed in a competing risk analysis considering death before d+100 as competing event and compared by Gray’s test. Complementary competing risk analysis was performed for cGVHD, which considered death within 12 months after HCT as competing event. Furthermore, the time-to-onset of all-grade aGVHD and aGVHD II-IV in the studied subgroups was calculated with the Kaplan-Meier method, obtaining event probabilities of time-to-event intervals. 1-year OS was analyzed *via* Kaplan-Meier analysis (35); subgroups were compared using the log-rank test; survival hazards were calculated by a Cox proportional hazards model (36). NRM and relapse were considered as competing events to each other and analyzed by competing risk analysis. P-values <0.05 were considered statistically significant. Clinical outcome analyses were done using the R (33) packages *survival (* 37), survminer (38) and cmprsk (39) (R version 3.6.3, R core Team, https://www.r-project.org/).

### Study approval

Study protocol approval was obtained by the institutional review board of the University Duisburg-Essen (Protocols N° 17-7675-BO and N° 18-8299-BO). All patients have given written informed consent to collection, electronic storage, and scientific analysis of anonymized HCT-specific patient data in accordance with German legislation and the revised Helsinki Declaration. We confirm that no patient can be identified through use of anonymized patient data.

## Results

### Patient characteristics

The HCT cohorts included in this study were balanced for age, sex, disease, graft source, conditioning and CMV recipient/donor serostatus (**Supplementary Table 3**). The sex mismatch proportion was lowest for MUD-PTCY patients (18%). Median study follow-up was 12 months.

### T cell manipulation with ATG or PTCy has comparable clinical efficacy at the cohort level

**Figure 1:**
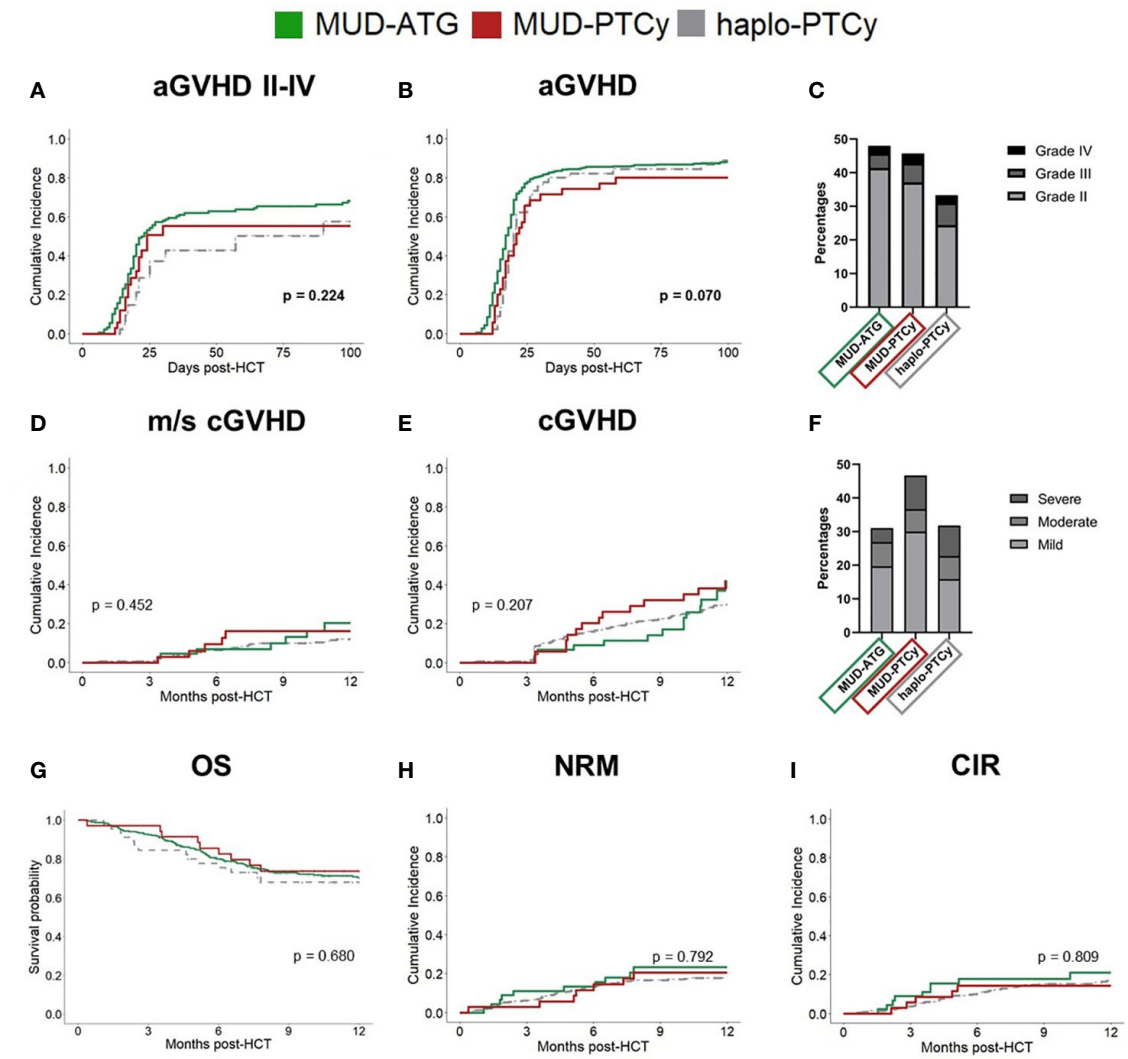
Comparable clinical outcomes in MUD patients receiving ATG or PTCy. Study cohorts: MUD-ATG (solid, green), MUD-PTCy (solid, red) and haplo-PTCy (dashed, grey). Cumulative incidence of **(A)** aGVHD (II-IV) and **(B)** all grade aGVHD within 100 days post-HCT. **(C)** Proportion of aGVHD grades within 100 days post-HCT in percent. Cumulative incidence of **(D)** moderate-severe cGVHD and **(E)** all grade cGVHD. **(F)** Proportion of cGVHD grades within 12 months post-HCT in percent. **(G)** Comparison of 12 months overall survival (OS) between study cohorts. **(H, I)** Cumulative Incidences of NRM and relapse. Equality of cumulative incidences functions (CIF’s) across the studied cohorts was compared by Gray’s test for competing risks. *P*-values < 0.05 were considered statistically significant. Given *p*-values refer to the comparison of MUD-ATG and MUD-PTCy patients.

At the cohort level, the 100-day cumulative incidence of grades II-IV and III-IV acute GVHD (aGVHD) did not significantly differ between MUD-ATG or MUD-PTCy (II-IV: MUD-ATG 68.3% and MUD-PTCy 55.3%, *p*=0.224; III-IV: MUD-ATG 19.8% and MUD-PTCy 17.2%, *p*=0.848; **Supplementary Table 4**, **Figures 1A, C**). All-grade aGVHD was numerically lower in the MUD-PTCy cohort (*p*=0.07, **Figure 1B**; **Supplementary Table 4**) and occurred relatively later (Median time 21 vs 17 days, p=0.058, **Supplementary Figure 2B**). Fine and Gray competing risk regression corroborated these results with lower all-grade aGVHD subdistribution hazards for MUD-PTCy (SHR 0.69, 95%CI 0.49-0.97, p=0.032, **Supplementary Table 5**), which did not retain significance for grades II-IV aGVHD (SHR 0.74, 95%CI 0.45-1.21, p=0.220, **Supplementary Table 6**). Both, all-grade chronic GVHD (cGVHD) and moderate-severe cGVHD at 12 months after HCT were comparable between both MUD cohorts (p=0.207 and p=0.452, **Figures 1E, D**). However, the incidence of severe cGVHD was numerically lower in the MUD-ATG cohort (**Figure 1F**). Overall survival (OS), non-relapse mortality (NRM) and the cumulative incidence of relapse (CIR) until 12 months did not differ between cohorts (**Figures 1G-I**, **Supplementary Tables 4**, **5**). Clinical outcomes with haplo-PTCy are detailed in **Supplementary Tables 5**–**7**.

### PTCy or ATG associate with the predominance of distinct T cell subsets

**Figure 2:**
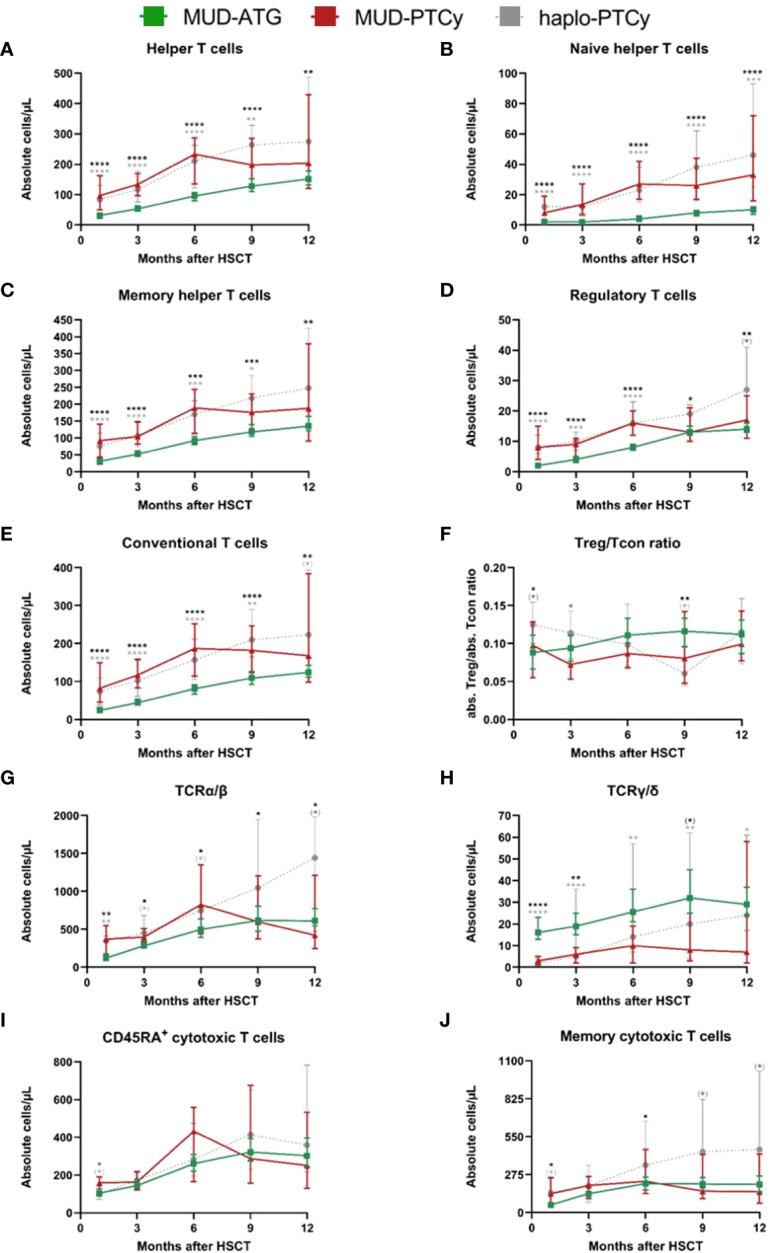
Significant differences in cellular immune reconstitution between MUD-PTCy and MUD-ATG patients: Increased helper T and regulatory T cell counts for PTCy patients and higher γδ-T cell counts in ATG patients. Immune reconstitution of T cell subsets within one year after HCT. T-lymphocyte subsets in the peripheral blood were characterized by multicolor flow cytometry. T helper cell subsets were gated on CD45^+^ cells and were identified as follows: **(A)** Helper T cells, CD3^+^/CD4^+^; **(B)** Naïve helper T cells, CD3^+^/CD4^+^/CD45RA^+^; **(C)** Memory helper T cells, CD3^+^/CD4^+^/CD45RO^+^. Furthermore the **(D)** regulatory T cells, CD3^+^/CD4^+^/CD25^+^/CD127^low^ and **(E)** conventional T cells, CD3^+^/CD4^+^/CD25^-^/CD127^high^ (not including the CD3+/CD4+/CD25-/CD127-low fraction) were gated among the CD3^+^/CD4^+^ cells. In **(F)** the ratio the Treg/Tcon ratio is shown. **(G, H)** illustrate the T cell receptor α/β, TCRα/β and T cell receptor γ/δ, TCRγ/δ positive T cells, respectively. **(I)** CD45RA^+^ cytotoxic T cells, CD3^+^/CD8^+^/CD45RA^+^; **(J)** Memory cytotoxic T cells, CD3^+^/CD8^+^/CD45RO^+^; These were gated within the CD3^+^ gate. Color codes for patient cohorts are the same as in **Figure 1**. Median absolute cell numbers were analyzed by the Mann-Whitney-U-test testing each group against the others at every time point. In the figure, only the p-values for the comparison between the MUD-ATG and MUD-PTCy group are illustrated. P-values < 0.05 were considered as statistically significant and are indicated with asterisks (*p* < 0.1, ^(^*^)^; *p* < 0.05, *; *p* < 0.01, **; *p* < 0.001, ***; and *p* < 0.0001, ****). All median values and sample numbers of the respective cohorts as well as the *p*-values are detailed in the online supplementary excel file.

Given the established role of T cells as aGVHD initiators, we investigated potential differences in immune reconstitution to detect alternative fractions involved in immune modulation by ATG or PTCy as T cell depletion and manipulation strategies. Indeed, despite the observed similar clinical efficacy (**Supplementary Table 4**) of both protocols, comparative analysis of cellular immune reconstitution revealed significant differences in T cell subsets. Throughout the first 12 months after HCT, ATG patients had significantly lower absolute counts within the helper T cell compartment (**Figures 2A–E**) compared to patients receiving PTCy. This pattern was also observed for TCR α/β T cells up to month 6 (**Figure 2G**). Interestingly, the absolute Treg counts were also significantly higher for the first 6 months after HCT in patients receiving PTCy as T cell manipulation (**Figure 2D**) compared to the MUD-ATG cohort. The Treg/Tcon ratio did not differ between the cohorts. In PTCy patients irrespective of the distinct donor type, early immune reconstitution up to month 6 was comparable confirming a PTCy specific benefit to the helper T cell compartment. Despite this early comparability of PTCy cohorts, median absolute counts of several helper T cell subsets stagnated in MUD-PTCy patients between months 6 and 12 (**Figure 2**) indicating a donor-type specific impact on helper T cell expansion after month 6. Contrary to the overall T cell cytopenia of the MUD-ATG cohort, its median TCR γ/δ T cell counts (**Figure 2H**) were significantly higher compared to MUD-patients receiving PTCy. The analysis of γδ T cell reconstitution further revealed broad confidence intervals for all analyzed subgroups, not originating from limited patient numbers as this was also observed for the large MUD-ATG cohort. Interestingly, the CD8^+^ subsets (**Figures 2I, J**) were not significantly affected by *in vivo* T cell depletion with ATG and did not account for differences between the MUD-ATG and MUD-PTCy cohorts. Of note, early NKT cell counts were also significantly higher in the MUD-ATG cohort (months 1 and 3, **Supplementary Figure 3F**). The reconstitution of further subsets e.g. cytotoxic- and activated T cells subsets (**Supplementary Figures 3B-E**), as well as NK- and B cells (**Supplementary Figures 3G–H**) was comparable between MUD-PTCy and MUD-ATG patients. Despite their early increase, CD3^+^ T cell levels in MUD-PTCy patients declined between months 6 and 12 leading to CD3^+^ numbers comparable to MUD-ATG patients at the end of the observation period. This finding was consistent throughout the majority of T cell subsets, with little exceptions, equalizing the above-described early differences in immune reconstitution of MUD patients between both T cell depleting regimens in the long-term.

### Time-series immune clustering dissects heterogeneity of phenotypes and outcomes within ATG and PTCy cohorts

**Figure 3:**
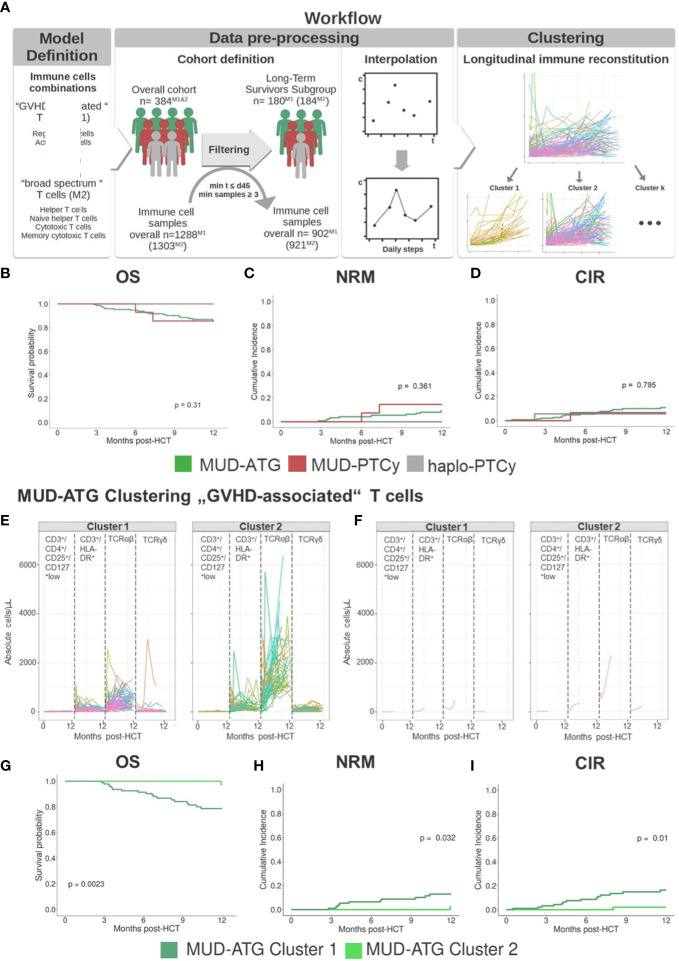
Time series clustering dissects heterogeneity of immune reconstitution data. **(A)** Depiction of time-series clustering workflow integrating the steps of data pre-processing, clustering, and clinical analysis. **(B-D)** Clinical outcome analysis for HCT patients (n=151 MUD-ATG, n=18 haplo-PTCy, and n=15 MUD-PTCy) that were included into time-series clustering approach: **(B)** Comparison of 12 months OS; cumulative incidences of **(C)** NRM and **(D)** relapse within 12 months post-HCT. **(E, F)** Individual patient immune cell data clustering in the MUD-ATG cohort using data of “GVHD-associated” T cells: CD3^+^/CD4^+^/CD25^+^/CD127^low^ regulatory T cells, CD3^+^/HLA-DR^+^ activated T cells, TCRα/β^+^ and TCR γ/δ^+^ T cells, The graph in **(E)** depicts each patients’ individual reconstitution pattern; **(F)** shows the most representative samples of each T cell subset calculated *via* partition around medoids (PAM). **(G-I)** Clinical outcome analysis for the MUD-ATG cohort using the cluster affiliation produced *via* time-series clustering. **(G)** Comparison of OS; cumulative incidences of **(H)** NRM and **(I)** relapse within 12 months post-HCT.

**Figure 4:**
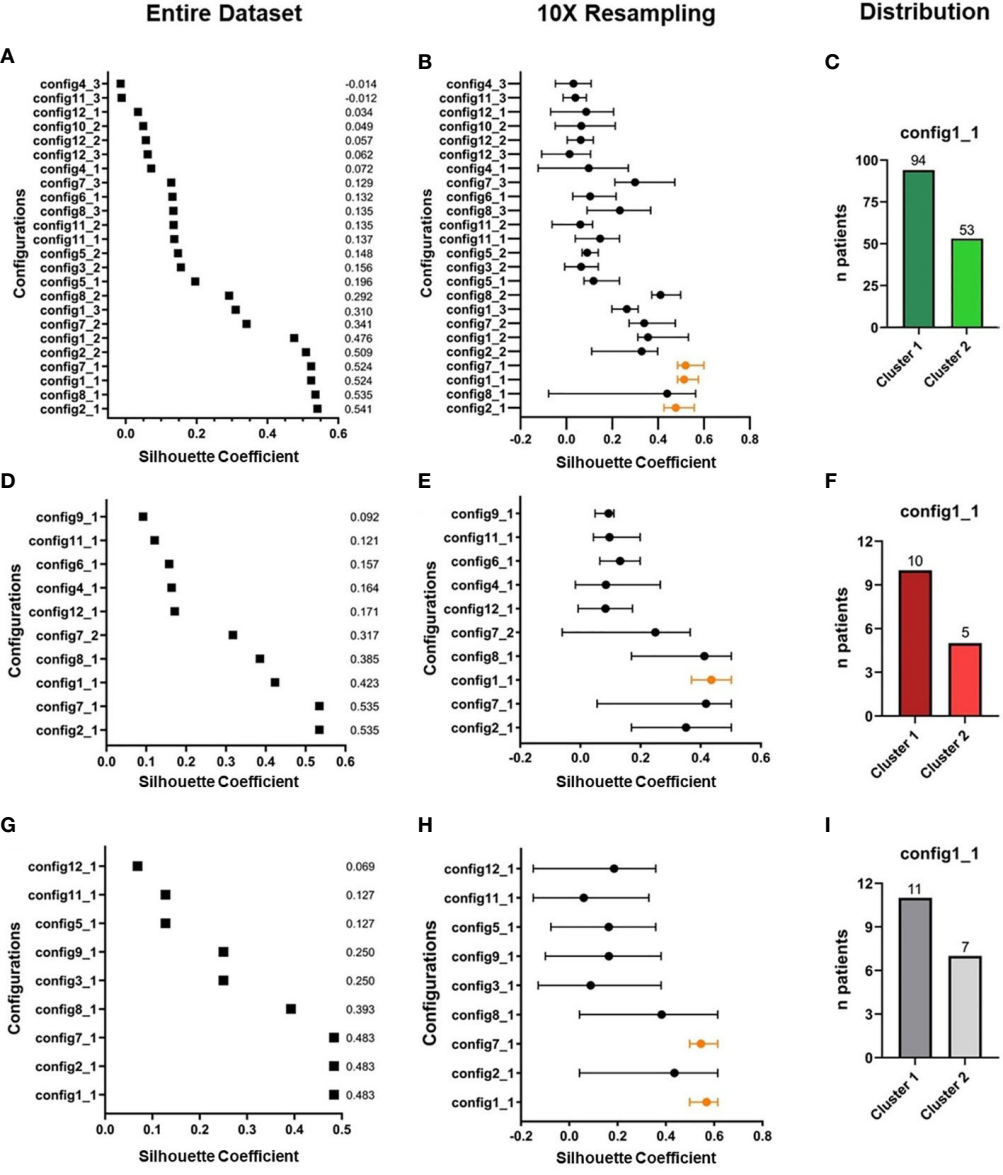
Overview of clustering results in the “GVHD-associated” T cell model. **(A, B)** Selection process of best parameter configurations. Among all possible clustering configurations **(A)** in the MUD-ATG dataset (*n*=147), the best are selected according to their respective silhouette coefficient (performance measure of the separation of subgroups during the clustering process, range from -1 to +1) for each configuration. **(B)** The silhouette coefficients are tested for robustness by 10x resampling. Only configurations, which have a high and robust silhouette coefficient, are considered for further analysis. **(C)** Proportion of MUD-ATG patients in each cluster of the best-performing clustering-configuration. **(D-F)** Selection of best parameter configurations in the **(D)** subset of MUD-PTCy patients (*n*=15) and **(E)** 10x resampling and **(F)** proportion of MUD-PTCy patients in each cluster of the best-performing clustering-configuration. **(G-I)** Selection of best parameter configurations in the **(G)** entire cohort of haplo-PTCy patients (*n*=18) and **(H)** in 10x resampling and **(I)** proportion of haplo-PTCy patients in each cluster of the best-performing clustering-configuration. Parameter configurations with concurrent good silhouette coefficients in the entire dataset and the most stable results in resampling are illustrated in orange.

**Figure 5:**
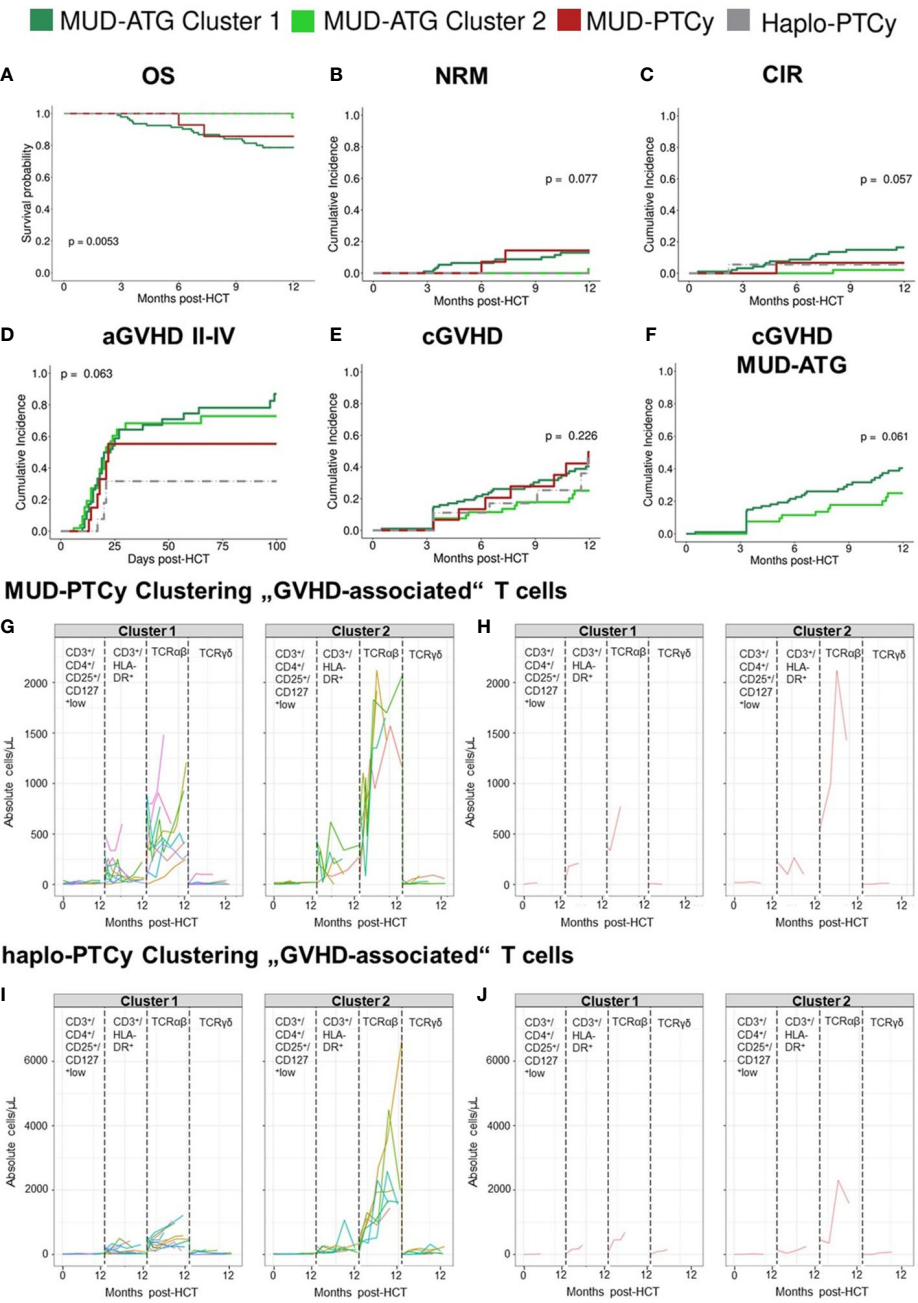
“GVHD-associated” T cell model identifies long-term survivors by their immune reconstitution patterns. **(A-E)** Clinical outcome analysis of all patients after time-series clustering of MUD-ATG patients in the “GVHD-associated” T cell model. **(A)** Comparison of OS; cumulative incidences of **(B)** NRM and **(C)** relapse within 12 months post-HCT; **(D)** cumulative incidence of aGVHD grades II-IV within 100 days post-HCT; **(E)** cumulative incidence of cGVHD. **(F)** Cumulative incidence of cGVHD in MUD-ATG patients only. **(G-J)** Individual patient immune cell data clustering in the **(G,H)** MUD-PTCy cohort and **(I, J)** haplo-PTCy cohort using data of “GVHD-associated” T cells: CD3^+^/CD4^+^/CD25^+^/CD127^low^ regulatory T cells, CD3^+^/HLA-DR^+^ activated T cells, TCRα/β^+^ and TCR γ/δ^+^ T cells, illustrated in distinct boxes. The graphs in **(G, I)** depict each patients’ individual reconstitution pattern in the respective subset; **(H, J)** show the most representative (medoid) samples of each subset calculated *via* partition around medoids (PAM).

**Figure 6:**
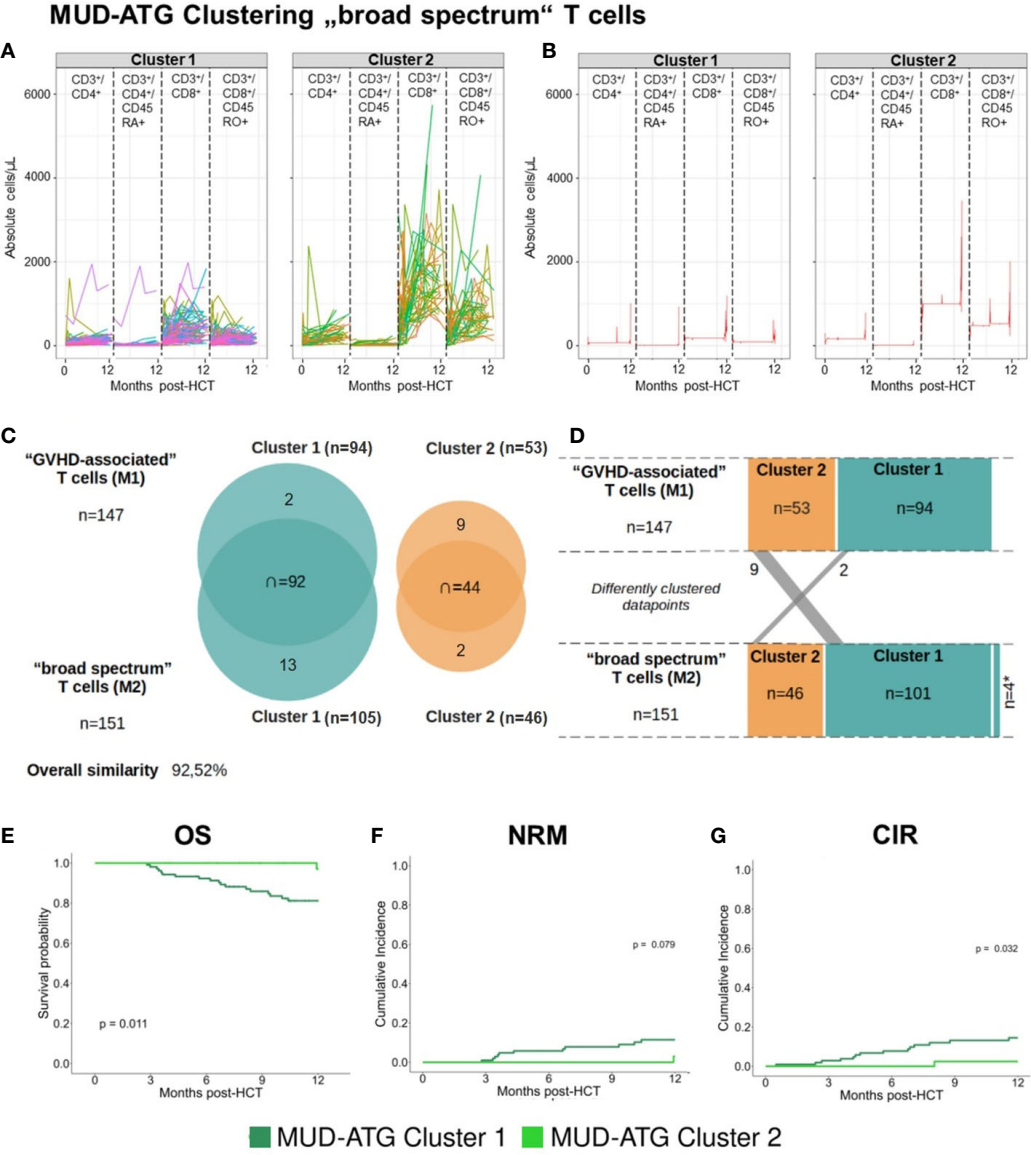
Time-series clustering of “broad spectrum” T cell subsets reveals comparable patient survival to the “GVHD-associated” T cell model. **(A, B)** Individual patient immune cell data clustering in the MUD-ATG cohort using data of “broad spectrum” T cells: CD3^+^/CD4^+^ helper T cells, CD3^+^/CD4^+^/CD45RA^+^ naïve helper T cells, CD3^+^/CD8^+^ cytotoxic T cells and CD3^+^/CD8^+^/CD45RO^+^ memory cytotoxic T cells, The graph in **(A)** depicts each patients’ individual reconstitution pattern in the respective subset; **(B)** shows the most representative (medoid) samples of each subset calculated *via* the prototype function DTW barycenter averaging (DBA). **(C)** Overlap between clusters of the “GVHD-associated”- and the “broad spectrum” T cell model. **(D)** Transition of patients between the clusters of both models. **(E-G)** Clinical outcome analysis for the MUD-ATG cohort using the cluster affiliation produced *via* the above shown time-series clustering. **(E)** Comparison of OS; cumulative incidences of **(F)** NRM and **(G)** of relapse within 12 months post-HCT.

**Figure 7:**
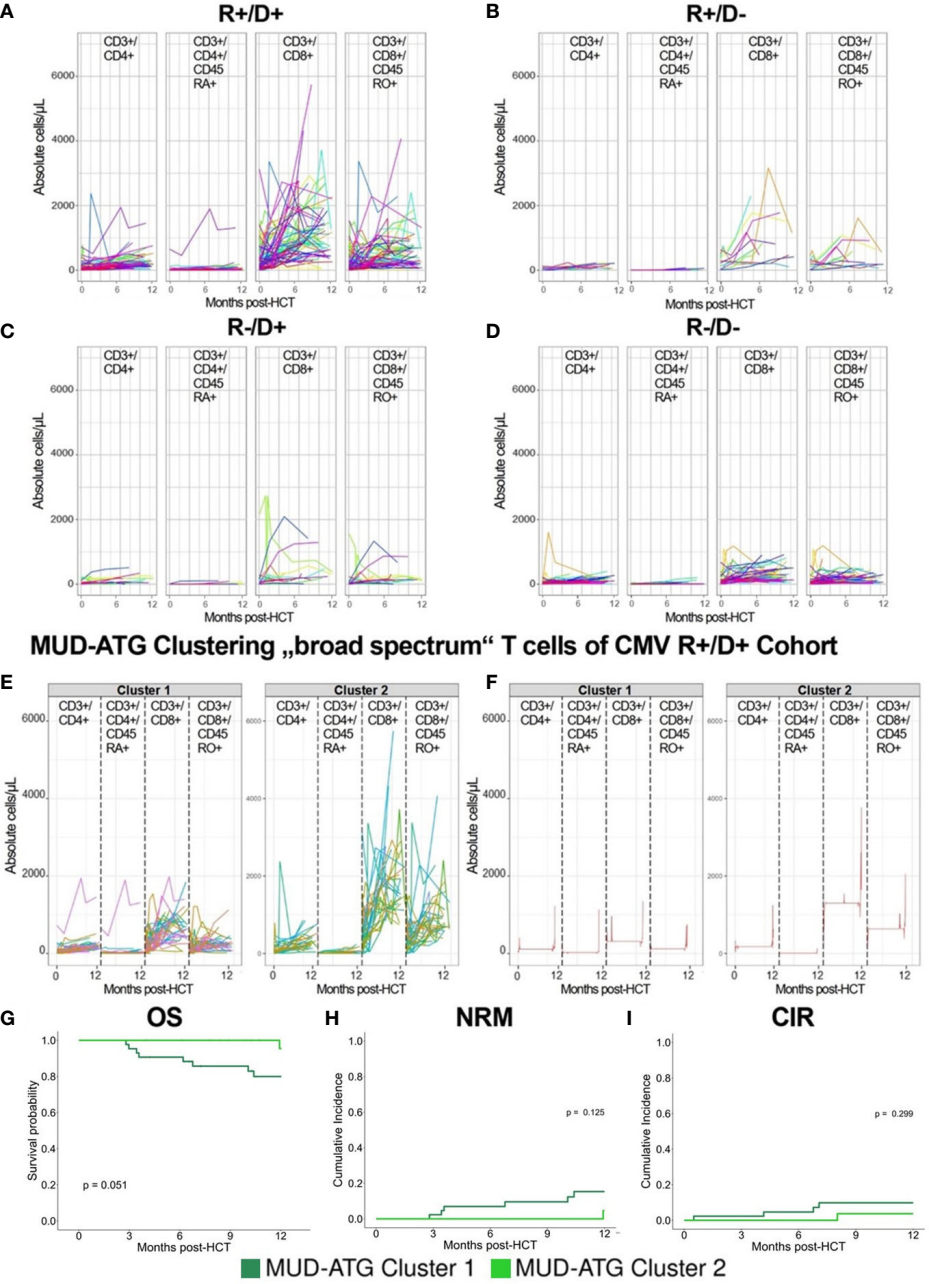
Clustering results of MUD-ATG patients in the “broad spectrum” T cell model dissected for CMV serostatus. **(A-D)** Individual patient immune cell data clustering of MUD-ATG patients using data of “broad spectrum” T cells (cells as in **Figure 6**) stratified by CMV recipient (R+) and donor (D+) serostatus. **(A)** CMV R+/D+, n=73 **(B)** CMV R+/D-, n=11, **(C)** CMV R-/D+, n=16 and **(D)** CMV R-/D-, n=51. **(E, F)** Individual patient immune cell data clustering in the **(G, H)** MUD-ATG R+/D+ subgroup time series clustering of T cell subsets as in the “broad spectrum” model. **(G-I)** Clinical outcome analysis for the MUD-ATG R+/D+ subset using the cluster affiliation produced *via* the time-series clustering from E-F. **(G)** Comparison of OS; cumulative incidences of **(H)** NRM and **(I)** of relapse within 12 months post-HCT.

Following the observation of broad confidence intervals in the pooled immune reconstitution data (e.g., in TCRγ/δ) and clinical outcomes, we hypothesized a relevant interindividual heterogeneity within each study cohort. Therefore, we analyzed cellular recovery with a data science approach able to dissect such heterogeneity within cohorts and to identify patients with similar reconstitution patterns, and possibly also homogenous clinical outcomes. Here, we developed two multi-dimensional parameter models integrating longitudinally measured reconstitution data of different T cell subsets for each patient. Because of the included cell types, we coined these models the “GVHD-associated”- and “broad spectrum” T cell model. Within each patient cohort (MUD-ATG, MUD-PTCy, and haplo-PTCy), both multi-dimensional models revealed distinct patterns by time-series clustering and dissected intra-cohort heterogeneity. Based on actual cell counts and reconstitution shapes, the models produced clusters, which, when correlated to clinical outcomes, revealed differences. The methodological workflow is detailed in the **Supplementary methods** and illustrated in **Figure 3A**. OS, NRM and CIR of patients included in this multi-dimensional analysis (n=180) were representative of patients with survival beyond d+100 in the overall cohort (**Figures 3B–D**), making a selection bias unlikely. Time-series clustering of T cell subsets from the “GVHD-associated” model dissected the MUD-ATG cohort into distinct patient subgroups (labelled patient clusters, **Figures 3E, F**). We determined the optimal model configuration *via* both a good and robust silhouette coefficient (configuration 1_1: *Sil*=0.524) as well as a balanced patient distribution (The MUD-ATG subgroup (n=147) was split into cluster 1: *n*=94 and cluster 2: *n*=43; **Figures 4A–C**). The cell subsets that contributed most to this clustering were activated- and αβ T cells, because they revealed greater differences in shape and higher absolute counts in both clusters over time as compared to Tregs and γδ T cells. Both of these T cell subsets were comparatively illustrated at smaller scales between the cohorts in **Supplementary Figure 6**. The corresponding cluster centroids (**Figure 3F**) confirmed distinct reconstitution shapes of each cluster. Patients in cluster 2, which had higher absolute counts of activated- and αβ T cells compared to cluster 1, had significantly lower NRM (*p*=0.032, **Figure 3H**) and relapse (*p*=0.01, **Figure 3I**) resulting in higher 1-year OS (98% vs. 79%, *p*=0.0023, **Figure 3G**). Next, we leveraged this cluster information comparing clinical outcomes between ATG clusters and the PTCy cohorts. Interestingly, patients from both the MUD-ATG cluster 1 and the MUD-PTCy cohort had similarly significantly decreased OS compared to MUD-ATG cluster 2 and haplo-PTCy (*p*=0.0053, **Figure 5A**). NRM and relapse (*p*=0.077 and *p*=0.057, respectively, **Figures 5B, C**) were numerically increased in MUD-ATG cluster 1. Despite a numerically lower incidence of aGVHD II-IV in MUD-PTCy patients (**Figure 5D**), their cGVHD incidence was comparable to MUD-ATG patients from cluster 1 (**Figure 5E**), which were both quantitatively higher compared to MUD-ATG cluster 2 (*p*=0.061, **Figure 5F**). We also tested the method of time-series clustering on the smaller PTCy cohorts using the “GVHD-associated” T cell model (**Figures 5G–J**). Again, the clustering successfully dissected intra-cohort heterogeneity in immune reconstitution (**Figures 5G–J**), although silhouette coefficients for optimal configurations were lower compared to the larger MUD-ATG cohort (**Figures 4D–I**). Similar to the results in the MUD-ATG cohort, the clustering of PTCy patients successfully distinguished two clusters in each setting characterized by relevant differences in absolute counts and reconstitution shape of activated- and αβ T cells (**Figures 5G–J**). A second clustering model integrating different “broad spectrum” T cell subsets, reflecting the pattern of CD4^+^ and CD8^+^ T cell reconstitution, also identified two separate clusters in the MUD-ATG cohort. Its optimal cluster configuration had a comparable silhouette coefficient (configuration 2_1: *Sil*=0.536) to the “GVHD-associated” T cell clustering model, and both good robustness and an appropriate patient distribution (cluster 1: *n*=105 and cluster 2: *n*=46; **Supplementary Figures 4A–C**). Here, the revealed clusters were most influenced by cytotoxic and memory cytotoxic T cells, which were higher in cluster 2 (**Figures 6A, B**). Strikingly, although both models integrated biologically different T cell subsets their degree of similarity was 92.5% as revealed by cluster model comparison (**Figure 6C**). Patient re-allocation between the two models was minimal (n=11, 7.5%, **Figure 6D**) and baseline characteristics were similar between the identified clusters e.g. for patient age or underlying disease (**Supplementary Tables 8**, **9**). Consequently, clinical analysis of these MUD-ATG clusters in the “broad-spectrum” T cell model revealed analog results to the “GVHD-associated” T cell model (**Figures 6E–G**), with the exception of a marginal significance for NRM. As reported above, the clustering of PTCy patients in the “broad spectrum” T cell model, also showed overall lower and more unstable silhouette coefficients (**Supplementary Figures 4D–I**). Again, cytotoxic T cell compartments appear to be the leading contributors to PTCy clusters (**Supplementary Figures 5A–D**). Given, the previously described impact of CMV serostatus on the T cell repertoire (40) we next examined the association between CMV serostatus and the time-series clustering. While the identified clusters in the configuration 2_1 had significantly distinct proportions of patients with R+ CMV serostatus, the differences in T cell kinetics between those with CMV R-/D-, CMV R-/D+ or CMV R+/D- serostatus were minimal (**Figures 7A–D**). However, the CMV R+/D+ subpopulation revealed important differences in T cell kinetics. We consequently tested if the clustering was further able to dissect T cell reconstitution within CMV R+/D+ patients and successfully applied the same cluster configuration 2_1 identifying relevant differences in T Cell kinetics (**Figures 7E, F**) as well as in OS (p=0.051, **Figure 7G**) within the CMV R+/D+ subgroup. Next, we tested if a combination of the most relevant T cell subsets from both the “GVHD-associated” and the “broad spectrum” T cell model would improve the clustering results. Indeed, the combination of CD3^+^/CD8^+^/CD45RO^+^, αβ T cells, HLA-DR^+^ T cells and CD3^+^ T cells (**Supplementary Figure 7**) associated with significant differences in clinical outcome and with slightly better separated OS curves (p=0.0014, **Supplementary Figure 7C**). Finally, we also tested a non-linear pre-processing approach instead of linear interpolation. The resulting curves had a similar, yet smoothened shape (**Supplementary Figures 8A, F**) and clinical outcome association was comparable (**Supplementary Figures 8C–E**). Taken together, both time-series clustering models integrating distinct T cell subsets were able to dissect intra-cohort heterogeneity in post-HCT immune reconstitution and to identify relevant patient subsets with distinct outcome.

## Discussion

T cell manipulating regimens, such as ATG or PTCy, are widely used to prevent GVHD in MUD-HCT, however individual patients benefit differentially. While larger studies compared their clinical effects, we focused on the cellular immune reconstitution and identified distinct T cell patterns in patients receiving ATG or PTCy. In PTCy patients, we found significantly higher regulatory T cell counts, while ATG patients had higher levels of γδ T cells. Despite these cellular differences, clinical outcomes were comparable indicating GVHD protection *via* distinct T cell subsets in patients with ATG or PTCy. As we observed some heterogeneity in longitudinal immune reconstitution data, we leveraged the data science approach of time-series clustering on multi-dimensional flow cytometry data, integrating data from both the actual T cell counts and its reconstitution shape. Within each cohort, we successfully identified two patient clusters with distinct reconstitution patterns, one of which associated with poor HCT outcomes, especially in the MUD-ATG setting. Most importantly, this clustering approach can be leveraged to dissect heterogeneity in cellular immune reconstitution patterns after HCT and support the identification and characterization of patient subgroups that are most likely to benefit from either platform of GVHD prophylaxis.

The clinical outcomes of our cohorts were within the expected range. The observed incidences of aGVHD II-IV in the ATG cohort were in line with the reported range from retrospective real world data studies (15, 16, 41–44), yet higher than in randomized trials (45, 46). Very recently, PTCy was shown to be more effective than ATG in preventing aGVHD II-IV in the unrelated donor setting (15), which is compatible with trends from our data. In our PTCy patients, the incidence of aGVHD II-IV was higher compared to previous studies (11, 15, 16), some of which used distinct calcineurin inhibitors (15). Nevertheless, aGVHD was comparable between MUD and haplo- settings as shown previously (11). In accordance with recent CIBMTR data (11), which showed similar relapse incidences in ATG and PTCy patients, this GVHD control (47) did not translate into increased relapse in our cohorts. For OS and NRM, our data confirmed previous findings (15, 42) of no statistical differences between MUD-ATG and MUD-PTCy patients.

Beyond these comparable clinical outcomes our data point to important differences in cellular immune reconstitution between both *in vivo* T cell manipulation regimens, showing a predominance of distinct T cell fractions, as well as relevant heterogeneity in patients within each cohort. These results complete the picture of the above-mentioned clinical studies, as cellular immune reconstitution of MUD-HCT patients receiving PTCy or ATG has not yet been extensively compared. Existing studies primarily focused on cohorts receiving either ATG or PTCy and controls without T cell depletion. ATG associated with a slow CD3^+^ T cell recovery and delayed (18, 19) dose-dependent recovery of CD4^+^ T cells (19). In contrast, PTCy had a sparing effect on regulatory T cells (20) enabling its preferential recovery after HCT (48). Two recent studies compared immune reconstitution after ATG or PTCy in mixed donor settings (e.g. combining data of MUD and haplo-HCT) both showing higher percentages of CD4^+^ T cells in the peripheral blood after PTCy. However, both studies exclusively examined early immune reconstitution until months 6 or 3 after HCT, respectively (16, 17). With MAC PTCy (16), only higher absolute counts of naïve CD4^+^ T cells were detected early after HCT, while patients with MAC ATG presented with consistently higher γδ T- and NKT cells through month 6. With RIC PTCy (17), NKT cell reconstitution was relatively lower compared to patients with ATG. Except for single months, no significant differences in absolute counts were detected in that study. Contrary to these studies, our analysis revealed significantly higher absolute counts of helper T cell subsets, especially of Tregs, in the MUD- and haplo-PTCy settings compared to MUD-ATG patients. In addition to such agent-specific effects our data provide evidence for a donor-type specific immune reconstitution distinguishing MUD-PTCy from haplo-PTCy patients. While early immune reconstruction did not differ significantly between each PTCy group, our data revealed higher T cell subset counts in haplo-HCT patients beyond month +6. This effect may have been blurred in previous analyses combining different donor types (16). Additionally, higher early total numbers of CD3^+^ T cells and cytotoxic T cells were seen after PTCy, but differences to ATG patients were not as pronounced as in helper T cells. The CD45RA^+^ cytotoxic T cell counts were comparable between MUD-ATG and MUD-PTCy recipients. Overall higher levels of T cells without increased aGVHD incidences in PTCy patients after HCT might be explained by the fact that PTCy does not eliminate alloreactive T cells, but instead leads to a functional impairment of these cells that can be sufficient to prevent differentiating donor T cells from causing GVHD (48). This may also be the case for αβ T cells, whose levels -early after HCT- were higher in PTCy patients than in MUD-ATG patients. Although αβ T cells have been associated with stronger alloreactive potential compared to γδ T cells (26), we did not observe increased aGVHD in our PTCy cohorts. The parallel increase in regulatory T cells, which have previously been reported to mediate aGVHD-protective effects (27), may explain this otherwise paradoxical finding of a numerically lower incidence of grades II-IV aGVHD. Both Tregs (48) and γδ T cells (49) can solidly expand after HCT. Higher γδ T cell counts in ATG patients early after transplant have now been reported from several centers (16, 19, 50) and likely relate to a preferential depletion of αβ T cells by polyclonal rabbit-anti-Jurkat T cell antibodies (Grafalon®). This Jurkat cell line has been previously described to express αβ but not γδ T cell receptors (51), which supports its contribution to ATG-based GVHD prophylaxis. PTCy patients, however, had lower γδ T cell levels without increased GVHD and instead higher regulatory T cell levels, supporting the hypothesis of distinct T cell subset expansions in each setting.

However, this canonical analysis of immune reconstitution focuses on the examination of one cell subset at a time not reflecting the interplay between distinct cellular subsets. Here, the use of median values may be efficient in providing an overview of cellular reconstitution (52) for specific patient subsets but are not very conclusive about the individual patient. This limitation may be overcome using the approach of time series clustering of multi-dimensional flow cytometry data, which to our knowledge has not been published before. This approach allows us to individually analyze cellular reconstitution within larger cohorts and exposes the heterogeneity within. An important asset of this method is that both the shape of immune reconstitution as well as the absolute cell counts are graphically displayed without any transformation and remain comprehensive for the user. Existing approaches to dissect the heterogeneity of cellular immune reconstitution from flow- or mass cytometry data are the viSNE (53) or UMAP (54) models or the principal component analysis (PCA) (55). Both viSNE and UMAP provide maps of clusters with similar patterns but do not show individual reconstitution curves, neither does PCA. As all these methods are assembled through dimensionality reduction steps, it makes the data less interpretable for physicians in their routine clinical use (56). Although the results from our multi-dimensional clustering approach, depended mainly on the cell counts, the reconstitution shape also contributed to the respective differentiations. Both of our T cell models pointed to specific T cell subsets, which dominated the clustering process, such as αβ T cells in the “GVHD-associated” model. Differences between the “conventional analysis” and the time series clustering approach were most pronounced in the distinct evaluation of αβ T cells. This gap results from the comparison of median values, which compensates for outliers whereas the time-series clustering integrates actual values on an individual basis. Time-series clustering was able to differentiate heterogeneous immune reconstitution patterns within each *in vivo* T cell manipulation platform. Indeed, this ability to distinguish smaller sets within larger patient cohorts and relate individual reconstitution patterns to clinical outcomes is its second asset. It is noteworthy that both analyzed T cell models robustly identified patients at risk for high NRM, although starting from distinct T cell subsets, which supports the importance of multi-dimensional T cell analysis. Lastly, time-series clustering also performs well and robust with limited sample number as exemplified in our PTCy subgroups. Yet, its strength to dissect T cell reconstitution of even small cohorts faces the limits of statistical comparability when comparing outcomes of cluster-defined subgroups with small patient numbers.

Despite the limitations of computational models related to data pre-processing requiring at least three consecutive flow cytometry samples and the missing functional assessment of the studied T cell subsets, the time series clustering efficiently dissected heterogeneity in immune reconstitution and clinical outcomes within the same T cell depletion and manipulation platform independent of analyzed pre- or peri-transplant factors. The limited availability of very early flow cytometry samples after HCT may have impaired the identification of aGVHD-specific signatures in both multi-dimensional T cell models, which may be overcome by more frequent biobanking. Hence, the current time series analysis results are best applicable to HCT patients with survival beyond day + 100. The studied antibody panel was limited. Cellular immune reconstitution following HCT may be impacted by a broad range of factors including GVHD, types and levels of immunosuppression, infectious events such as CMV and relapse. In particular, CMV exposure results in a strong imprinting on T cell diversity (57, 58) and also impacted the absolute T cell counts during post HCT immune reconstitution in our study. Hence, time-series-clustering of T cells resulted in distinct proportions of CMV R+ patients in its clusters, which was best visible in CMV R+/D+ patients (**Figure 7**). However, that same clustering successfully dissected immune reconstitution within these CMV R+/D+ patients and identified relevant subsets within, showing that this analytic approach may be successfully adjusted for CMV-dependent bias of T cell reconstitution.

In conclusion, using the analysis of cellular reconstitution patterns we show that GVHD protection appears to be driven by different T cell subsets in patients receiving either PTCy or ATG for GVHD prophylaxis, namely regulatory T cells or γδ T cells, respectively. Leveraging time series clustering on T cell reconstitution, we dissected the heterogenous cellular immune reconstitution landscape of these cohorts and thereby identified individuals with poor outcomes after HCT based on their immune reconstitution profiles.

## Data availability statement

The source code has been deposited in GitHub and may be obtained from the corresponding author upon request. To enable independent replication of our methods, we included detailed descriptions of preprocessing and model development in the methods section and in the supplementary material. Upon reasonable request, de-identified primary data can be provided in accordance with ethics restrictions.

## Ethics statement

The studies involving human participants were reviewed and approved by the University of Duisburg-Essen. Written informed consent for participation was not required for this study in accordance with the national legislation and the institutional requirements by the Institutional Review Board of the University.

## Author contributions

ATT, SL, and TG designed the study. MF, UB, and SL performed flow cytometry. SL, RB, and ATT collected data. MF, UB, and NL participated in data acquisition. SL and TG performed model development and statistical analysis. AT supervised research. ATT, SL, TG, EA-B, KF, and DB interpreted the data. RB and NL participated in data analysis. ATT, DB, RB, HCR, and NL provided clinical expertise. ATT and SL wrote the manuscript. EA-B, DB, UB, NL, TG, and KF contributed to write the manuscript. All authors had access to primary clinical trial data, read and approved the final manuscript.
