# Supplementary material for: Time series clustering of T cell subsets dissects heterogeneity in immune reconstitution and clinical outcomes among MUD-HCT patients receiving ATG or PTCy
Source: Front Immunol. 2023 Mar 20;14:1082727. doi: 10.3389/fimmu.2023.1082727 (PMC10067907; doi:10.3389/fimmu.2023.1082727)
Supplement: Supplementary file 1 [file DataSheet_1.pdf]

Supplementary Material to

Time series clustering of T cell subsets dissects heterogeneity in immune reconstitution and clinical outcomes among MUD-HCT patients receiving ATG or PTCy

Saskia Leserer[1, 2, 3], Theresa Graf[1, 2], Rashit Bogdanov[1, 2], Martina Franke[1], Ulrike Buttkereit[1], Esteban Arrieta-Bolanos3, 4, Nils Leimkühler[1], Katharina Fleischhauer[3, 4], H. Christian Reinhardt[1,5], Dietrich W. Beelen[1] and Amin T. Turki[1, 2, 3]

[1] Department of Hematology and Stem Cell Transplantation, West-German Cancer Center, University Hospital Essen, Essen, Germany

2 Computational Hematology Lab, Department of Hematology and Stem Cell Transplantation, West-German Cancer Center, University Hospital Essen, Essen, Germany

3 Institute for Experimental Cellular Therapy, West-German Cancer Center, University Hospital Essen, Essen, Germany

4 German Cancer Consortium (DKTK). Site Essen/Düsseldorf

5 Cancer Center Cologne Essen (CCCE)

Supplementary Methods:

Time-series clustering of immune cell reconstitution

Time-series clustering was used to classify and visualize longitudinal patient-specific T cell reconstitution data into structures with maximal similarity. Time-series clustering utilizes a specific distance measure, called dynamic time warping (DTW), to transform longitudinal time-series datasets into machine-readable data for clustering algorithms[1,2]. DTW can compare time series, including those that are shifted in time, and identify similarities in shape[1,2]. The DTW algorithm creates a local cost matrix to find an optimal warping path (=minimal distance) between two time series[2]. For subsequent partitional clustering following DTW, time series prototypes are included as essential factor, as the resulting prototypes function as cluster centroids resembling the average time series of a cluster[2]. The number of clusters, *k*, also defines the number of randomly initialized centroids[2]. The distance between all series within the data as well as all centroids is determined, and each time series is ascribed to its closest centroid's cluster[2]. T**Fehler! Textmarke nicht definiert.**hese steps are repeated until reaching

the defined iteration limit or until constant clusters are built[2]. The DTW barycenter averaging and the partition around medoids[2] are the only time-series prototypes that can be used in the context of DTW. Internal validation of the clustering algorithms' performance can be evaluated by internal cluster validity indices, such as the silhouette coefficient (*Sil*)[2,3]. This silhouette coefficient ranges between -1 to +1, positive *Sil* values indicate an efficient separation of clusters reaching the *Sil* optimum[3] at +1.

Time-series clustering was applied to a series of three different multi-dimensional T cell models consisting of 1) "GVHD-associated" T cells: $CD3^+/CD4^+/CD25^+/CD127^+$low regulatory T cells, $CD3^+$/HLA-DR^+ activated T cells, TCRα/β^+ and TCR γ/δ^+ T cells and 2) "broad spectrum" T cells: $CD3^+/CD4^+$ helper T cells, $CD3^+/CD4^+/CD45RA^+$ naïve helper T cells, $CD3^+/CD8^+$ cytotoxic T cells and $CD3^+/CD8^+/CD45RO^+$ memory cytotoxic T cells. An additional "combined" T cell model was composed of the most relevant subsets of the previous models consisting of $CD3^+/CD8^+/CD45RO^+$ memory cytotoxic T cells, $CD3^+$/HLA-DR^+ activated T cells, TCRα/β^+ T cells and $CD3^+$ T cells. Longitudinal immune reconstitution data were pre-processed by filtering of the dataset and an interpolation of individual patient data points. Inclusion criteria were a) ≥ 3 available flow cytometry sampling timepoints within the first 12 months after HCT and b) first flow cytometry measurement $\leq$ d+45 post-HCT to have a representative sample collection. Next, data gaps between discrete data points were filled by linear interpolation described as follows

(1)

where ( ) and ( ) are two known values that describe the cell concentration *c* on the respective day *t*. Linear interpolation between all known values results in longitudinal data with daily intervals. Due to technical reasons relating to the clustering algorithm code, the time series were shifted to the offset of *t*=0. For the distance measurement by DTW such shift is negligible. After data pre-processing n=147 patients remained in the MUD-ATG cohort ("GVHD-associated" T cell model, n=151 "broad spectrum" T cell model) as well as n=15 and n=18 in the MUD-PTCy or haplo-PTCy cohorts, respectively. DTW was applied on the interpolated data and the resulting distance matrix was used for data clustering. In this study, we tested two different algorithms, the partitional and the hierarchical clustering. Initial experiments using the hierarchical clustering showed acceptable performance results, but suffered from dysbalanced patient distribution within the clusters and required inappropriate computational costs. For the final model development we therefore focused on the partitional clustering. Here, we performed a hyperparameter tuning integrating the following features: pre-defined cluster numbers (k=2, k=3, k=4), methods for distance measurement (DTW or DTWbasic), prototype functions and z-normalization, if present. The common transformation formula is described as follows

(2)

where i N, x_i is the original datapoint, μ is the time series mean and σ the standard deviation.

In total, 36 different feature combinations were analyzed, and the best-performing setting was evaluated by comparing the silhouette coefficient of all possible combinations (Suppl. Table 2). To identify the most robust model by internal validation, we performed a 10-fold data resampling of random 2/3 datasets to evaluate the robustness of each configuration by the variability of silhouette coefficients within the resampling. The final cluster configurations were selected according to the following quality criteria: a) high silhouette coefficient, b) the robustness of the configuration and c) sufficient patient distribution within the clusters. Given its larger size, time-series clustering as a method to analyze immune cell reconstitution was developed utilizing the MUD-ATG cohort dataset and was later applied to both smaller PTCy subgroups. The performance of time series clustering remained high despite differences in dataset size.

For dissecting the imprint of CMV serostatus on the results of time-series clustering, we performed a further analysis on the MUD-ATG cohort with the "broad spectrum" T cell model. Patients were ordered by CMV Serostatus (n=73 R+/D+, n=11 R+/D-, n=16 R-/D+ and n=51 R-/D-). Time series T cell data of these CMV serogroups were interpolated as described in the main methods section and an additional partitional clustering of the MUD-ATG "broad spectrum" T cells R+/D+ subgroup was performed to test the potential of time-series clustering under the impact of CMV exposure (Suppl. Fig 7E-F)

References

1. Aghabozorgi S, Seyed Shirkhorshidi A, Ying Wah T. Time-series clustering – A decade review. *Information Systems*. 2015;53:16-38.
2. Sardá-Espinosa A. Time-Series Clustering in R Using the dtwclust Package. *The R Journal*. 2019;11:22.
3. Rousseeuw PJ. Silhouettes: A graphical aid to the interpretation and validation of cluster analysis. *Journal of Computational and Applied Mathematics*. 1987;20:53-65.

Supplementary Tables

Supplementary Table 1: Surface markers and antibody references for immune reconstitution monitoring via flow cytometry analysis

Antibody	Clone	Isotype	Label	Manufacturer	Reference Number
CD45	J33	IgG1	KrO	Coulter	B36294
CD3	UCHT1	IgG1	PB	Coulter	A93687
CD4	13B8.2	IgG1	APC750	Coulter	A94682
CD8	B9.11	IgG1	APC	Coulter	IM2469
CD45RA	ALB11	IgG1	FITC	Coulter	AO7786
CD45RO	UCHL1	IgG2a	ECD	Coulter	B49192
CD25	B1.49.9	IgG2a	PE	Coulter	A07774
CD19	J3-119	IgG1	PC5.5	Coulter	B49211
CD127	R34.34	IgG1	PC7	Coulter	A64618
CD314 (NKG2D)	ON72	IgG1	PE	Coulter	A08934
HLA-DR	Immu-357	IgG1	PC5.5	Coulter	B20024
CD16	3G8	IgG1	PC7	Coulter	6607118
CD56	N901(NKH-1)	IgG1	PC7	Coulter	A21692
CD14	RMO52	IgG2a	APC750	Coulter	A86052
TCR α/β		IgG1	APC	Miltenyi	130-113-527
TCR γ/δ	11F2	IgG1	FITC	Miltenyi	130-113-503

Supplementary Table 2: Summary of studied clustering configurations. Parameter tuning: 36 different clustering configurations. Additional parameters for *maximal iteration, norm distance and norm centroid* (not shown) were uniformly set to 30, L1 and L1, respectively.

Config ID	k	preproc	center_preproc	distance	centroid
config1_1	2	none		dtw	pam
config1_2	3	none		dtw	pam
config1_3	4	none		dtw	pam
config2_1	2	none		dtw	dba
config2_2	3	none		dtw	dba
config2_3	4	none		dtw	dba
config3_1	2	zscore	FALSE	dtw	pam
config3_2	3	zscore	FALSE	dtw	pam
config3_3	4	zscore	FALSE	dtw	pam
config4_1	2	zscore	FALSE	dtw	dba
config4_2	3	zscore	FALSE	dtw	dba
config4_3	4	zscore	FALSE	dtw	dba
config5_1	2	zscore	TRUE	dtw	pam
config5_2	3	zscore	TRUE	dtw	pam
config5_3	4	zscore	TRUE	dtw	pam
config6_1	2	zscore	TRUE	dtw	dba
config6_2	3	zscore	TRUE	dtw	dba
config6_3	4	zscore	TRUE	dtw	dba
config7_1	2	none		dtw_basic	pam
config7_2	3	none		dtw_basic	pam
config7_3	4	none		dtw_basic	pam
config8_1	2	none		dtw_basic	dba
config8_2	3	none		dtw_basic	dba
config8_3	4	none		dtw_basic	dba
config9_1	2	zscore	FALSE	dtw_basic	pam
config9_2	3	zscore	FALSE	dtw_basic	pam
config9_3	4	zscore	FALSE	dtw_basic	pam
config10_1	2	zscore	FALSE	dtw_basic	dba
config10_2	3	zscore	FALSE	dtw_basic	dba
config10_3	4	zscore	FALSE	dtw_basic	dba
config11_1	2	zscore	TRUE	dtw_basic	pam
config11_2	3	zscore	TRUE	dtw_basic	pam
config11_3	4	zscore	TRUE	dtw_basic	pam
config12_1	2	zscore	TRUE	dtw_basic	dba
config12_2	3	zscore	TRUE	dtw_basic	dba
config12_3	4	zscore	TRUE	dtw_basic	dba

Abbreviations: *Config ID*, name of the configuration. *k*, number of clusters. *Preproc*, describes the normalization of time series data before clustering. If none, no normalization was performed, if z-score, z-normalization was performed. *Center_preproc*, if centering was performed during z-normalization. *Distance*, two different DTW distance measurements were used: dtw and dtw_basic. *Centroid*, describes whether pam (partitioning around medoids) or dba (dynamic time warping barycenter averaging) has been set as centroid.

Supplementary Table 3: Patient baseline characteristics for cohorts with either ATG or PTCy as GVHD prophylaxis.

Characteristics	MUD-ATG		MUD-PTCy		haplo-PTCy		p
	n	%	n	%	n	%	
Total enrolled and treated	304	80	35	9	45	12	
Median age at transplantation (range)	59	(19-77)	56	(20-70)	56	(18-78)	0.249
Male sex	170	56	23	66	28	62	0.430
Disease							0.754
Acute myeloid leukemia	147	48	16	46	23	51	
Myelodysplastic syndromes	40	13	3	9	4	9	
Myeloproliferative neoplasia	27	9	4	11	2	4	
Acute lymphoblastic leukemia	26	9	4	11	7	16	
Chronic myeloid leukemia	16	5	1	3	1	2	
Chronic myelomonocytic leukemia	8	3	0	0	0	0	
Non-Hodgkin's lymphoma	29	10	4	11	4	9	
Multiple myeloma	1	0	1	3	1	2	
Aplastic Anemia	3	1	1	3	1	2	
Other hematologic disorders	7	2	1	3	2	4	
Graft source							0.307
PBSC	302	99	34	97	45	100	
BM	2	1	1	3	0	0	
HLA-matching/Donor Type							**<0.0001**
MUD	304	100	35	100	0	0	
Haploidentical	0	0	0	0	45	100	
Conditioning							0.167
Myeloablative conditioning	204	67	18	51	28	62	
Reduced intensity conditioning	100	33	17	49	17	38	
TBI containing	120	39	11	31	19	42	0.587
GVHD Prophylaxis							**<0.0001**
CsA	0	0	0	0	1	2	
CsA + MTX	254	84	0	0	0	0	
CsA + MMF	11	4	0	0	0	0	
TAC + MMF	25	8	35	100	44	98	
Other	14	5	0	0	0	0	
Recipient/Donor sex constellation							**<0.0001**
Female/Female	71	23	9	26	10	22	
Male/Male	152	50	20	57	15	33	
Female/Male	63	21	3	9	7	16	
Male/Female	18	6	3	9	13	29	
CMV Serology							0.132
R+/D-	34	11	9	26	8	18	
R+/D+	141	46	12	34	23	51	
R-/D+	28	9	5	14	4	9	
R-/D-	101	33	9	26	10	22	

Abbreviations: *ATG*, anti-T-lymphocyte globulin; *BM*, bone marrow; *CMV*, cytomegalovirus; *CsA*, cyclosporin A; *D*, donor; *haplo*, haploidentical; *MMF*; mycophenolate mofetil; *MTX*, methotrexate; *MUD*, matched unrelated donor; *n*, number of cases; *p*; p-value; *PBSC*, peripheral blood stem cells; *PTCy*, post-transplant cyclophosphamide; *R*, recipient; *TBI*, total body irradiation.

Supplementary Table 4. Comparison of cumulative incidences of relevant outcomes between the MUD-ATG and MUD-PTCy cohorts: 100-day incidence for aGVHD (II-IV and III-IV) and 12 months post-HCT for cGVHD (moderate-severe), relapse and NRM .

	Cumulative Incidences				
	MUD-ATG		MUD-PTCy		*p*
	%	95% CI [%]	%	95% CI [%]	
**Acute GVHD (all grades)**	88.2	84.6 – 91.9	80.0	67.8 – 94.4	0.070
**Acute GVHD II-IV**	68.3	61.4 – 76.0	55.3	39.4 – 77.6	0.224
**Acute GVHD III-IV**	19.8	12.3 – 31.9	17.2	5.5 – 53.4	0.848
**Chronic GVHD (all grades)**	29.7	24.7 – 35.7	41.4	27.7 – 62.0	0.207
**Chronic GVHD (moderate-severe)**	12.1	8.6 – 17.0	16.2	7.2 – 36.2	0.452
**Relapse**	16.9	13.0 – 21.9	14.4	6.4 – 32.4	0.809
**NRM**	18.2	14.3 – 23.3	20.5	10.6 – 39.7	0.792

Abbreviations: *CI,* Confidence interval.
Death within 100 days or 12 months post-HCT were regarded as competing event for acute or chronic GVHD, respectively. Relapse and NRM were considered competing events to each other.

Supplementary Table 5: Univariate competing risk regression analysis of clinical outcomes. Acute GVHD outcomes at 100-days, all other outcomes at 12 months post-HCT.

**Outcome**	**Competing risk regression**		
	**SHR**	**95% CI**	***p***
Acute GVHD			
MUD-ATG	—	—	—
MUD-PTCy	0.69	0.49 – 0.97	**0.032**
Haplo-PTCy	0.77	0.60 – 1.00	**0.050**
Acute GVHD II-IV			
MUD-ATG	—	—	—
MUD-PTCy	0.74	0.45 – 1.21	0.220
Haplo-PTCy	0.54	0.33 – 0.86	**0.010**
Chronic GVHD			
MUD-ATG	—	—	—
MUD-PTCy	1.47	0.86 – 2.51	0.160
Haplo-PTCy	1.04	0.58 – 1.84	0.900
Chronic GVHD (moderate-severe)			
MUD-ATG	—	—	—
MUD-PTCy	1.44	0.56 – 3.71	0.450
Haplo-PTCy	1.17	0.46 – 2.99	0.740
Overall Survival			
MUD-ATG	—	—	—
MUD-PTCy	0.87	0.44 – 1.72	0.684
Haplo-PTCy	1.17	0.67 – 2.06	0.582
Relapse			
MUD-ATG	—	—	—
MUD-PTCy	0.90	0.35 – 2.29	0.820
Haplo-PTCy	1.36	0.66 – 2.80	0.410
NRM			
MUD-ATG	—	—	—
MUD-PTCy	1.11	0.52 – 2.41	0.780
Haplo-PTCy	1.31	0.67 – 2.56	0.430

Abbreviations: *CI,* Confidence interval; S*HR,* subdistribution hazard ratio; —, reference group.
Overall survival was analyzed with Cox proportional hazard regression yielding Hazard ratios (HR). Death and relapse were regarded as competing events towards acute and chronic GVHD. Relapse and NRM were competing events to each other.

Supplementary Table 6. Competing risk regression analysis of pre-transplant factors for the outcome of grades II-IV aGVHD.

**Covariate**	**Competing risk regression**		
	**SHR**	**95% CI**	***p***
Subgroup			
MUD-ATG	—	—	—
MUD-PTCy	0.73	0.44 – 1.21	0.220
haplo-PTCy	0.54	0.33 – 0.86	**0.010**
Disease			
AML	—	—	—
others	0.97	0.73 – 1.30	0.840
Recipient Age			
<50 years	—	—	—
≥50 years	1.13	0.80 – 1.59	0.500
Recipient Sex			
Male	—	—	—
Female	0.99	0.74 – 1.33	0.940
Donor Age			
<30 years	—	—	—
≥30 years	0.98	0.73 – 1.30	0.870
Donor Sex			
Male	—	—	—
Female	0.91	0.66 – 1.25	0.570
Conditioning			
MAC	—	—	—
RIC	1.03	0.76 – 1.40	0.850
Total body irradiation			
Yes	—	—	—
No	0.93	0.69 – 1.25	0.610
ECOG			
0-1	—	—	—
2-3	0.72	0.38 – 1.34	0.300
CMV Serology			
R-/D-	—	—	—
R-/D+	0.56	0.30 – 1.04	0.066
R+/D-	0.93	0.57 – 1.50	0.760
R+/D+	1.00	0.72 – 1.38	1.000

Abbreviations: *CI,* Confidence interval; S*HR,* subdistribution hazard ratio; —, reference group.
Death was regarded as competing event towards aGVHD grades II-IV.

Supplementary Table 7: Comparison of 100-day cumulative incidences of aGVHD (II-IV and III-IV) and 12-months cumulative incidences of cGVHD (moderate-severe), relapse and NRM between the MUD-PTCy and haplo-PTCy subgroups.

	Cumulative Incidences				
	MUD-PTCy		Haplo-PTCy		p
	%	95% CI [%]	%	95% CI [%]	
**Acute GVHD**	80.0	67.8 – 94.4	88.9	80.2 – 98.6	0.565
**Acute GVHD II-IV**	55.3	39.4 – 77.6	57.6	38.9 – 85.2	0.372
**Acute GVHD III-IV**	17.2	5.5 – 53.4	18.7	6.8 – 51.4	0.968
**Chronic GVHD**	41.4	27.7 – 62.0	41.8	27.5 – 63.3	0.594
**Chronic GVHD (moderate-severe)**	16.2	7.2 – 36.2	20.3	10.3 – 40.0	0.941
**Relapse**	14.4	6.4 – 32.4	20.9	11.6 – 37.7	0.490
**NRM**	20.5	10.6 – 39.7	23.2	13.5 – 40.2	0.740

Abbreviations: CI, Confidence interval.
Death within 100 days and 1-year post-HCT were regarded as competing event towards acute and chronic GVHD, respectively. Relapse and NRM were considered competing events to each other.

Supplementary Table 8: Patient baseline characteristics from MUD-ATG patients in cluster 2 of the "GVHD-associated" T cell model and cluster 2 from the "broad spectrum" T cell model.

**Characteristics**	**"GVHD-associated" T Cells, Cluster 2**		**"broad spectrum" T Cells, Cluster 2**		***p***
	**n**	**%**	**n**	**%**	
Total enrolled and treated	53	100	46	100	
Median age at transplantation (range)	61	(20-73)	61,5	(20-73)	0.838
Male sex	18	34	17	37	0.756
Disease					>0.999
Acute myeloid leukemia	33	62	30	65	
Myelodysplastic syndromes	8	15	6	13	
Myeloproliferative neoplasia	3	6	2	4	
Acute lymphoblastic leukemia	3	6	3	7	
Chronic myeloid leukemia	0	0	0	0	
Chronic myelomonocytic leukemia	1	2	1	2	
Non-Hodgkin's lymphoma	2	4	2	4	
Multiple myeloma	0	0	0	0	
Aplastic Anemia	1	2	1	2	
Other hematologic disorders	2	4	1	2	
Graft source					>0.999
PBSC	53	100	46	100	
BM	0	0	0	0	
Conditioning					0.843
Myeloablative conditioning	31	58	26	57	
Reduced intensity conditioning	22	42	20	43	
TBI containing	16	30	15	33	0.796
GVHD Prophylaxis					0.823
CsA	0	0	0	0	
CsA + MTX	48	91	42	91	
CsA + MMF	1	2	1	2	
TAC + MMF	3	6	3	7	
Other	1	2	0	0	
Recipient/Donor sex constellation					0.983
Female/Female	17	32	15	33	
Male/Male	16	30	15	33	
Female/Male	18	34	14	30	
Male/Female	2	4	2	4	
CMV Serology					0.970
R+/D-	6	11	5	11	
R+/D+	42	79	37	80	
R-/D+	3	6	3	7	
R-/D-	2	4	1	2	

Abbreviations: *ATG*, anti-T-lymphocyte globulin; *BM*, bone marrow; *CMV*, cytomegalovirus; *CsA*, cyclosporin A; *D*, donor; *haplo*, haploidentical; *MMF*; mycophenolate mofetil; *MTX*, methotrexate; *MUD*, matched unrelated donor; *n*, number of cases; *p*; p-value; *PBSC*, peripheral blood stem cells; *PTCy*, post-transplant cyclophosphamide; *R*, recipient; *TBI*, total body irradiation.

Supplementary Table 9: Patient baseline characteristics from MUD-ATG patients in cluster 1 of the "GVHD-associated" T cell model and cluster 1 from the "broad spectrum" T cell model.

Characteristics	"GVHD-associated" T Cells, Cluster 1		"broad spectrum" T Cells, Cluster 1		*p*
	n	%	n	%	
Total enrolled and treated	94	100	105	100	
Median age at transplantation (range)	56	(19-75)	56	(19-75)	0.864
Male sex	56	60	60	57	0.728
Disease					>0.999
Acute myeloid leukemia	45	48	50	48	
Myelodysplastic syndromes	6	6	8	8	
Myeloproliferative neoplasia	11	12	12	11	
Acute lymphoblastic leukemia	12	13	12	11	
Chronic myeloid leukemia	8	9	9	9	
Chronic myelomonocytic leukemia	2	2	3	3	
Non-Hodgkin's lymphoma	7	7	7	7	
Multiple myeloma	0	0	0	0	
Aplastic Anemia	1	1	1	1	
Other hematologic disorders	2	2	3	3	
Graft source					>0.999
PHSC	94	100	105	100	
BM	0	0	0	0	
Conditioning					0.888
Myeloablative conditioning	69	73	78	74	
Reduced intensity conditioning	25	27	27	26	
TBI containing	42	45	46	44	0.902
GVHD Prophylaxis					0.963
CsA	0	0	0	0	
CsA + MTX	83	88	91	87	
CsA + MMF	1	1	2	2	
TAC + MMF	6	6	7	7	
Other	4	4	5	5	
Recipient/Donor sex constellation					0.959
Female/Female	22	23	24	23	
Male/Male	51	54	55	52	
Female/Male	16	17	21	20	
Male/Female	5	5	5	5	
CMV Serology					0.934
R+/D-	5	5	6	6	
R+/D+	30	32	36	34	
R-/D+	10	11	13	12	
R-/D-	49	52	50	48	

Abbreviations: *ATG*, anti-T-lymphocyte globulin; *BM*, bone marrow; *CMV*, cytomegalovirus; *CsA*, cyclosporin A; *D*, donor; *haplo*, haploidentical; *MMF*; mycophenolate mofetil; *MTX*, methotrexate; *MUD*, matched unrelated donor; *n*, number of cases; *p*; p-value; *PBSC*, peripheral blood stem cells; *PTCy*, post-transplant cyclophosphamide; *R*, recipient; *TBI*, total body irradiation.

Supplementary Table 10: Flow cytometry sample statistics

	MUD-ATG		MUD-PTCy		MUD-ATG vs. MUD-PTCy		haplo-PTCy		MUD-PTCy vs. haplo-PTCy		MUD-ATG vs. haplo-PTCy	
	n	median	n	median	*p*		n	median	*p*		*p*	
T cells												
Month 1	233	151	24	380	**0.034**	*	23	350	0.740		**0.024**	*
Month 3	293	313	44	405.5	0.140		31	459	0.887		0.179	
Month 6	194	529.5	25	849	0.153		29	850	0.670		**0.033**	*
Month 9	178	649	21	628	0.815		27	1123	0.120		0.064	(*)
Month 12	145	676	13	467	0.709		17	1488	**0.048**	*	**0.040**	*
Helper T cells												
Month 1	233	32	23	97	**<0.0001**	****	23	84	0.652		**<0.0001**	****
Month 3	293	53	44	134	**<0.0001**	****	32	117	0.956		**<0.0001**	****
Month 6	194	95.5	25	233	**<0.0001**	****	29	211	0.683		**<0.0001**	****
Month 9	179	128	21	198	**0.007**	**	27	264	0.111		**<0.0001**	****
Month 12	145	152	13	204	0.122		17	275	0.160		**0.002**	**
Cytotoxic T cells												
Month 1	233	97	23	232	0.081	(*)	23	205	0.896		**0.043**	*
Month 3	293	232	44	310	0.489		32	285	0.744		0.371	
Month 6	194	421	25	668	0.366		29	566	0.585		0.093	(*)
Month 9	179	491	21	395	0.784		27	821	0.170		0.169	
Month 12	145	474	13	311	0.476		17	946	0.065	(*)	0.076	(*)
NK cells												
Month 1	233	222	23	237	0.611		23	180	0.258		0.348	
Month 3	293	192	44	162.5	0.321		32	206	0.693		0.702	
Month 6	194	201	25	182	0.462		29	186	0.783		0.551	
Month 9	179	214	21	146	0.137		27	176	0.715		0.303	
Month 12	145	182	13	172	0.676		17	155	0.813		0.908	
B cells												
Month 1	233	9	23	1	**<0.0001**	****	23	1	0.567		**<0.0001**	****
Month 3	293	15	44	7	**0.005**	**	32	6	0.523		0.251	
Month 6	194	40.5	25	36	0.789		29	52	0.456		0.361	
Month 9	179	95	21	42	0.608		27	69	0.769		0.662	
Month 12	145	132	13	137	0.979		17	142	0.592		0.427	
Activated T cells												
Month 1	233	38	23	103	**0.011**	*	23	109	0.605		**0.025**	*
Month 3	292	83	44	96.5	0.274		32	102.5	0.655		0.669	
Month 6	194	96.5	25	126	0.515		29	155	0.780		0.196	
Month 9	179	101	21	91	0.273		27	150	**0.046**	*	0.140	
Month 12	145	84	13	55	0.407		17	148	0.142		0.172	
CD45RA+ cytotoxic T cells												
Month 1	233	105	23	159	0.052	(*)	23	100	**0.025**	*	0.936	
Month 3	293	144	44	163	0.815		32	171.5	0.744		0.500	
Month 6	194	260.5	25	432	0.386		29	284	0.853		0.487	
Month 9	179	322	21	287	0.846		27	414	0.536		0.517	
Month 12	145	303	13	251	0.379		17	359	0.110		0.255	
Memory cytotoxic T cells												
Month 1	233	55	23	137	0.058	(*)	23	144	0.952		**0.025**	*
Month 3	293	137	44	197	0.749		32	195	0.636		0.370	
Month 6	194	210.5	25	227	0.639		29	344	0.195		**0.025**	*
Month 9	179	208	21	156	0.335		27	441	0.089	(*)	0.128	
Month 12	145	204	13	152	0.492		17	457	0.095	(*)	0.111	
Naïve helper T cells												
Month 1	233	2	23	8	**<0.0001**	****	23	12	0.909		**<0.0001**	****
Month 3	293	2	44	13.5	**<0.0001**	****	32	12	0.704		**<0.0001**	****
Month 6	194	4	25	27	**<0.0001**	****	29	23	0.488		**<0.0001**	****
Month 9	179	8	21	26	**<0.0001**	****	27	38	0.487		**<0.0001**	****
Month 12	145	10	13	33	**0.0004**	***	17	46	0.241		**<0.0001**	****
Memory helper T cells												
Month 1	233	31	23	92	**<0.0001**	****	23	76	0.620		**<0.0001**	****
Month 3	293	52	44	104	**<0.0001**	****	32	105.5	0.931		**<0.0001**	****
Month 6	194	92	25	189	**0.0003**	***	29	170	0.780		**0.0002**	***
Month 9	179	118	21	176	**0.045**	*	27	218	0.131		**0.0002**	***
Month 12	145	135	13	188	0.228		17	248	0.229		**0.009**	**
NKT cells												
Month 1	233	10	23	2	**<0.0001**	****	23	2	0.265		**<0.0001**	****
Month 3	293	10	44	3	**<0.0001**	****	32	3	0.619		**<0.0001**	****
Month 6	194	12	25	7	**0.030**	*	29	12	0.358		0.334	
Month 9	179	15	21	7	**0.007**	**	27	11	0.581		**0.029**	*
Month 12	145	15	13	7	0.096	(*)	17	13	0.179		0.872	
Conventional T cells												
Month 1	233	24	23	82	**<0.0001**	****	23	74	0.613		**<0.0001**	****
Month 3	293	44	44	117	**<0.0001**	****	32	102.5	0.923		**<0.0001**	****
Month 6	194	81	25	187	**<0.0001**	****	29	157	0.621		**<0.0001**	****
Month 9	179	109	21	182	**0.006**	**	27	210	0.192		**<0.0001**	****
Month 12	145	124	13	168	0.092	(*)	17	223	0.229		**0.002**	**
Regulatory T cells												
Month 1	232	2	23	8	**<0.0001**	****	23	8	0.516		**<0.0001**	****
Month 3	293	4	44	9	**0.0004**	***	32	10	0.203		**<0.0001**	****
Month 6	194	8	25	16	**<0.0001**	****	29	16	0.887		**<0.0001**	****
Month 9	179	13	21	13	0.507		27	19	0.361		**0.037**	*
Month 12	145	14	13	17	0.264		17	27	0.084	(*)	**0.001**	**
Treg/Tcon ratio												
Month 1 (‡)	213	0.088	23	0.098	0.590		23	0.125	0.051	(*)	**0.050**	*
Month 3 (‡)	286	0.094	44	0.072	0.268		31	0.114	**0.028**	*	0.258	
Month 6	194	0.111	25	0.087	0.346		29	0.098	0.651		0.734	
Month 9	179	0.117	21	0.081	0.064	(*)	27	0.061	0.295		**0.002**	**
Month 12	145	0.112	13	0.099	0.676		17	0.115	0.483		0.979	
TCR α/β												
Month 1	223	123	23	373	**0.005**	**	23	348	0.553		**0.006**	**
Month 3	288	283.5	44	397.5	0.084	(*)	31	451	0.742		**0.043**	*
Month 6	194	498.5	25	826	0.074	(*)	29	747	0.786		**0.022**	*
Month 9	179	617	21	600	0.670		27	1048	0.110		**0.039**	*
Month 12	145	613	13	423	0.840		17	1443	0.059	(*)	**0.042**	*
TCR γ/δ												
Month 1	223	16	23	3	**<0.0001**	****	23	2	0.307		**<0.0001**	****
Month 3	288	19	44	6	**<0.0001**	****	31	5	0.942		**0.007**	**
Month 6	194	25.5	25	10	**0.001**	**	29	14	0.241		0.176	
Month 9	179	32	21	8	**0.001**	**	27	20	0.280		0.095	(*)
Month 12	145	29	13	7	**0.011**	*	17	24	0.103		0.744	

(‡): Values of n are lower for month 1 and 3 of the Treg/Tcon ratio as there were cases for which the absolute cells/µL were 0 in both subsets.

Supplementary Figures

A

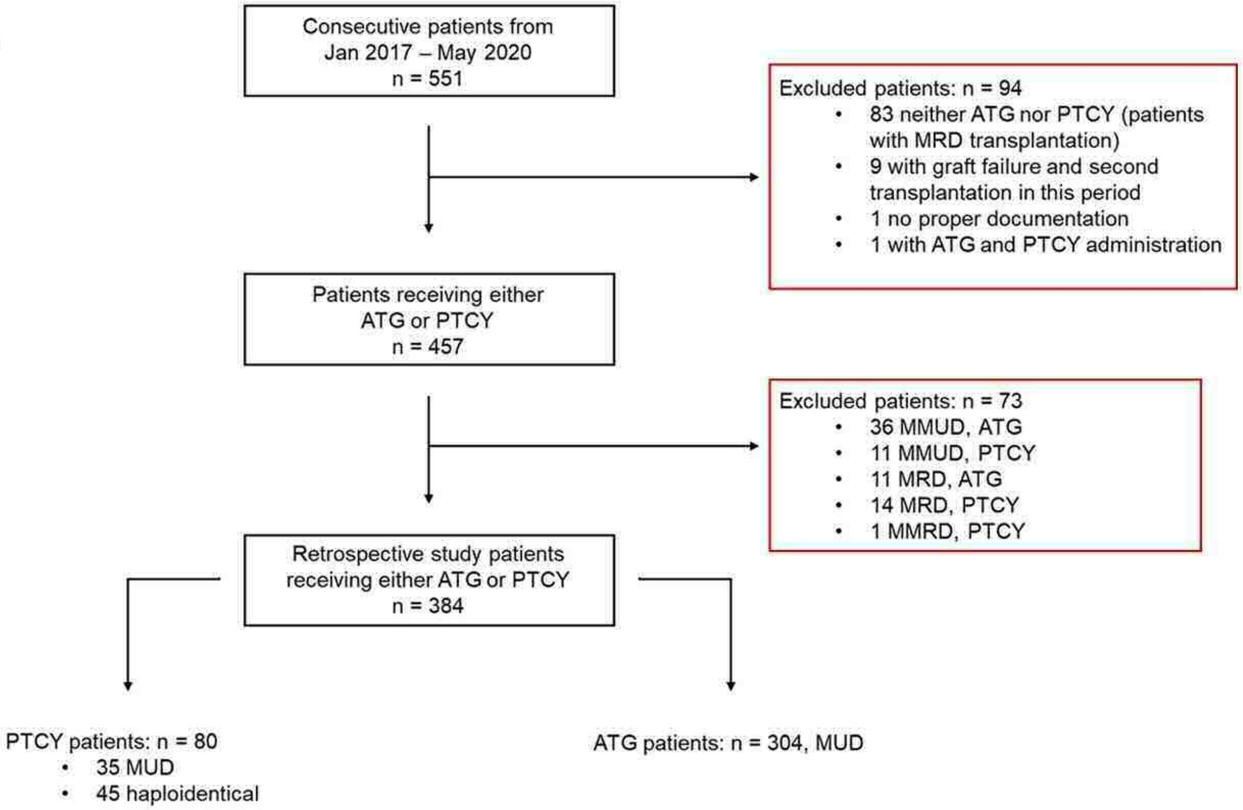

B

	Month 1 (n=)	Month 3 (n=)	Month 6 (n=)	Month 9 (n=)	Month 12 (n=)
MUD-ATG	233	293	194	178	145
MUD-PTCy	23	44	25	21	13
haplo-PTCy	23	32	29	27	17

Supplementary Figure 1. A: CONSORT flow diagram for selection of study cohort. All 551 patients with HCT between January 2017 and May 2020 were screened. For study inclusion, the following selection criteria were applied: allogeneic HCT from haploidentical- or matched unrelated donors (MUD) with either ATG or PTCY as GVHD prophylaxis. **B**: **Overview on flow cytometry sampling per time point and cohort.**

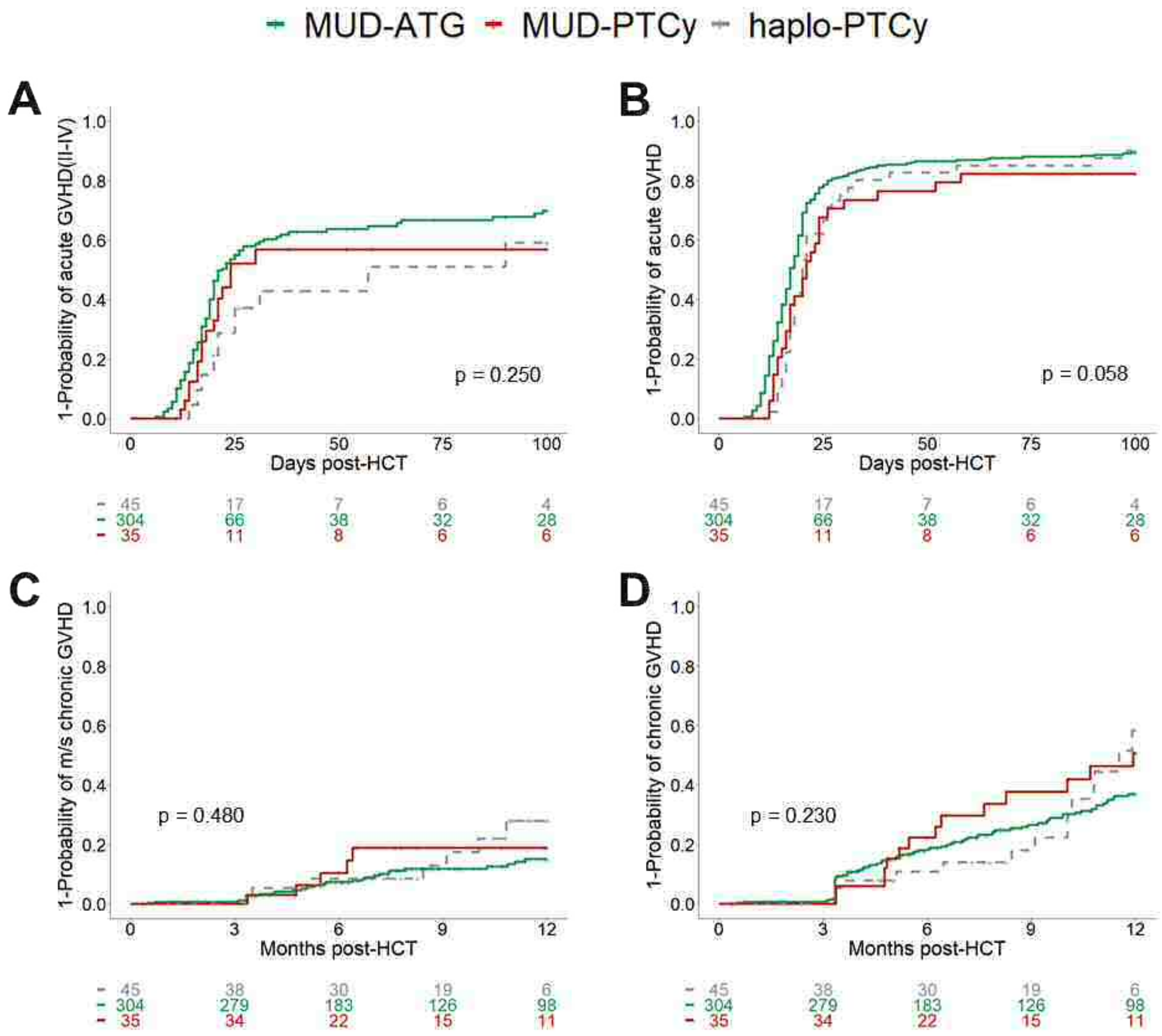

Supplementary Figure 2. Time to acute or chronic GVHD did not significantly differ between MUD-ATG and MUD-PTCy patients. Studied patients comprise the following cohorts: MUD-ATG (solid, green), MUD-PTCy (solid, red) and haplo-PTCy (dashed, grey). **(A, B)** Time to acute GVHD II-IV and acute GVHD (all grades) within 100 days post-HCT calculated with the Kaplan-Meier method. **(C, D)** Time to moderate-severe chronic GVHD and chronic GVHD (all grades) within 12 months post-HCT, as obtained by Kaplan-Meier method. *P*-values < 0.05 were considered as statistically significant. Given *p*-values refer to the comparison of MUD-ATG and MUD-PTCy cohorts.

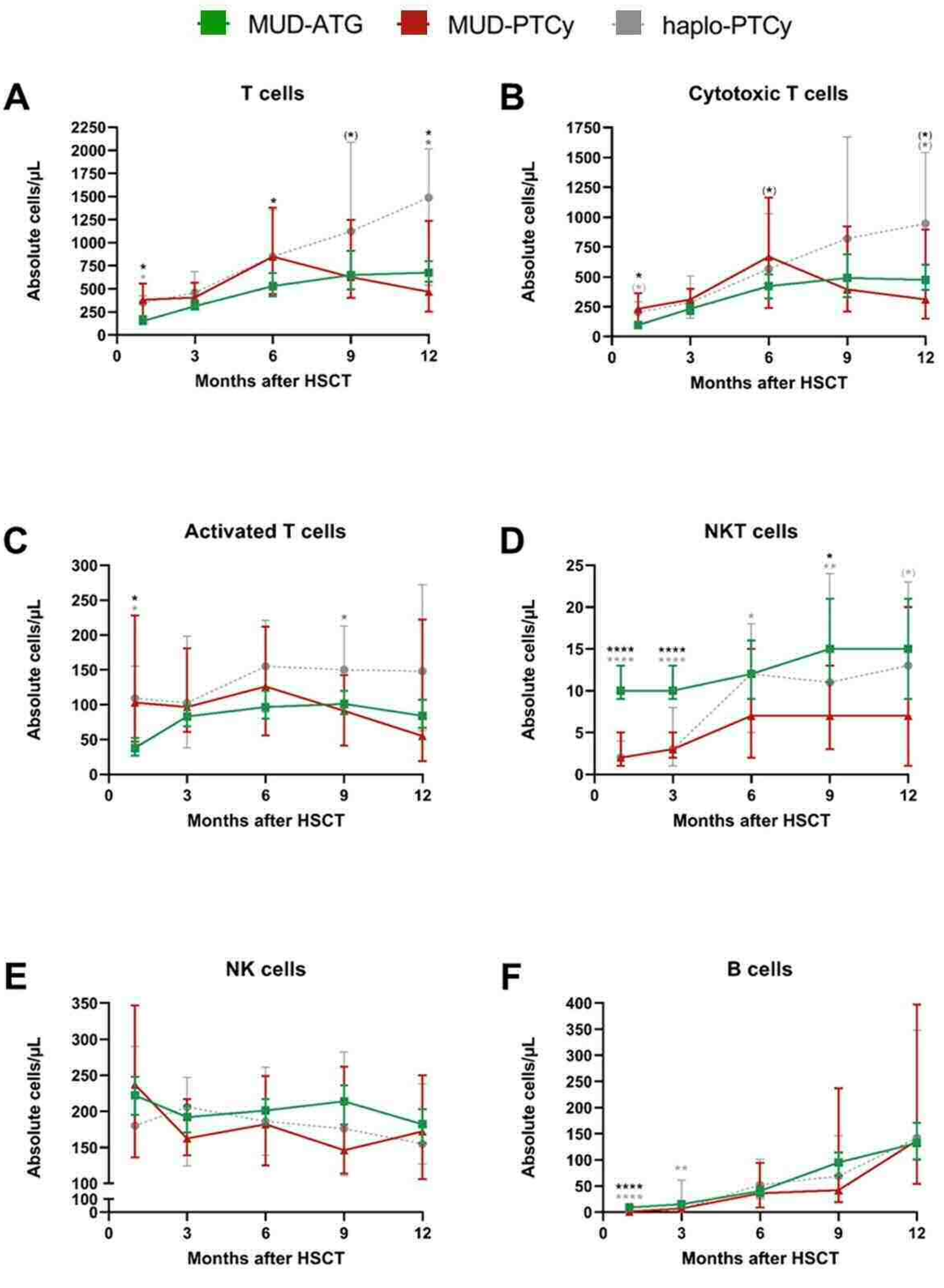

Supplementary Figure 3. Reconstitution of cytotoxic T cell subsets, NK and B cells. Immune reconstitution 12 months after HCT. T cell subsets were gated within the CD45$^+$ gate as follows: **(A)** T cells, CD3$^+$; **(B)** Cytotoxic T cells, CD3$^+$/CD8$^+$; **(C)** Activated T cells, CD3$^+$/HLA-DR$^+$; **(D)** NKT

cells, CD3$^+$/CD16$^+$/CD56$^+$/CD314$^+$; **(E)** NK cells, CD16$^+$/CD56$^+$/CD314$^+$; **(F)** B cells, CD19$^+$. Color codes for cohorts are the same as in Suppl. Figure 2. Median absolute cell numbers and the 95% CI were analyzed by the Mann-Whitney-U-test. In the figure, only the p-values for the comparison between the MUD-ATG and MUD-PTCy group are illustrated. P-values < 0.05 were considered as statistically significant and are indicated with asterisks ($p < 0.1$, $^{(*)}$; $p < 0.05$, *; $p < 0.01$, **; $p < 0.001$, ***; and $p < 0.0001$, ****). Median values and sample numbers of the respective cohorts as well as the *p*-values are summarized in the attached excel file.

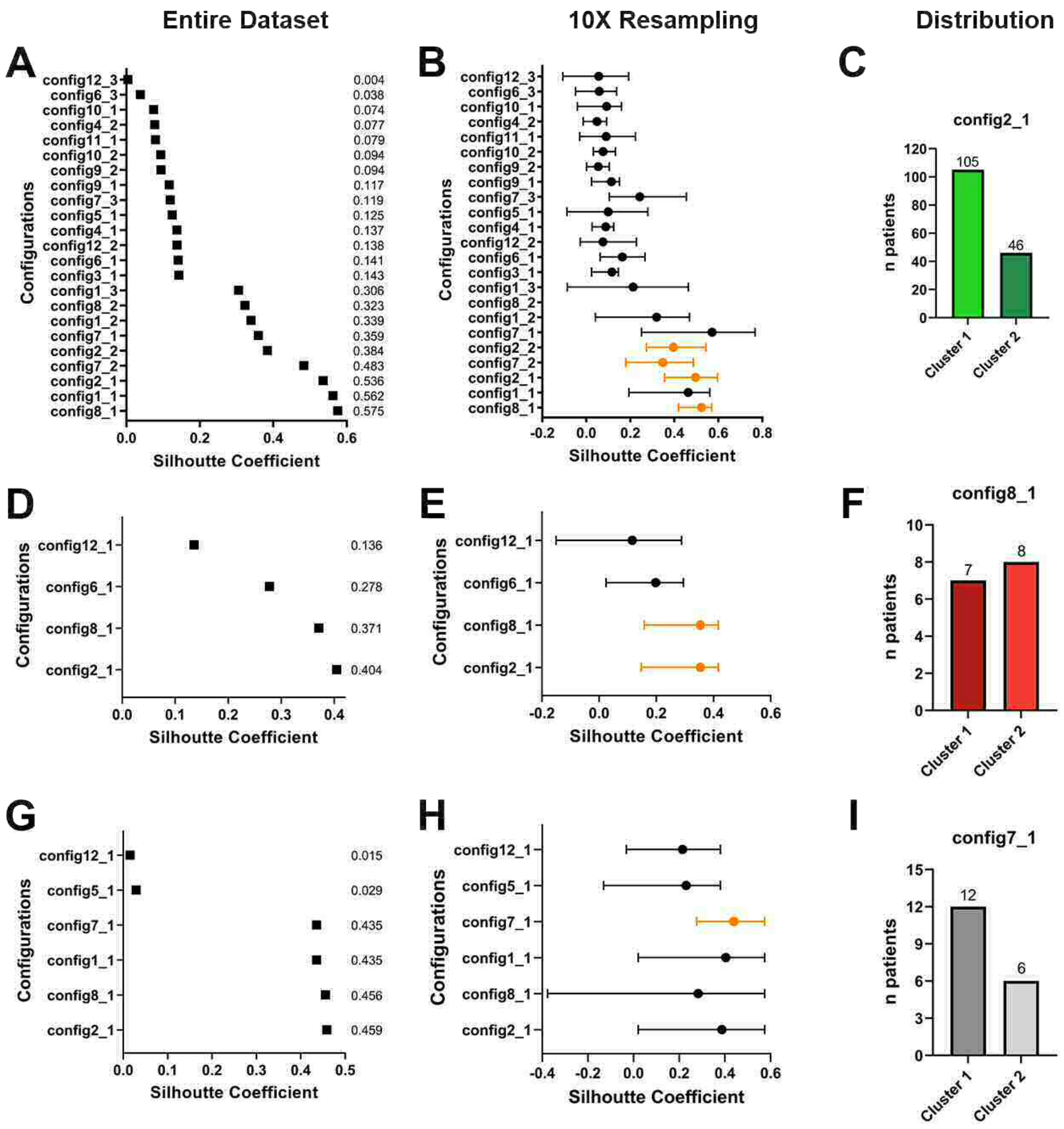

Supplementary Figure 4. Overview of clustering results in the "broad spectrum" T cell model. (A-B) Selection of parameter configurations **(A)** in the entire MUD-ATG dataset (*n*=151) with their respective silhouette coefficients and **(B)** results from 10x resampling for robustness testing. **(C)** Distribution of MUD-ATG patients within the selected configuration. **(D-F)** Selection of parameter configurations in the **(D)** entire cohort of MUD-PTCy patients (*n*=15) and **(E)** in 10x resampling and **(F)** patient distribution within the selected parameter configuration. **(G-I)** Selection of parameter configurations in the **(G)** entire cohort of haplo-PTCy patients (*n*=18) and **(H)** in 10x resampling and **(I)** patient distribution within the selected parameter configuration. Parameter configurations with concurrent good silhouette coefficients in the entire dataset and the most stable results in resampling are illustrated in orange.

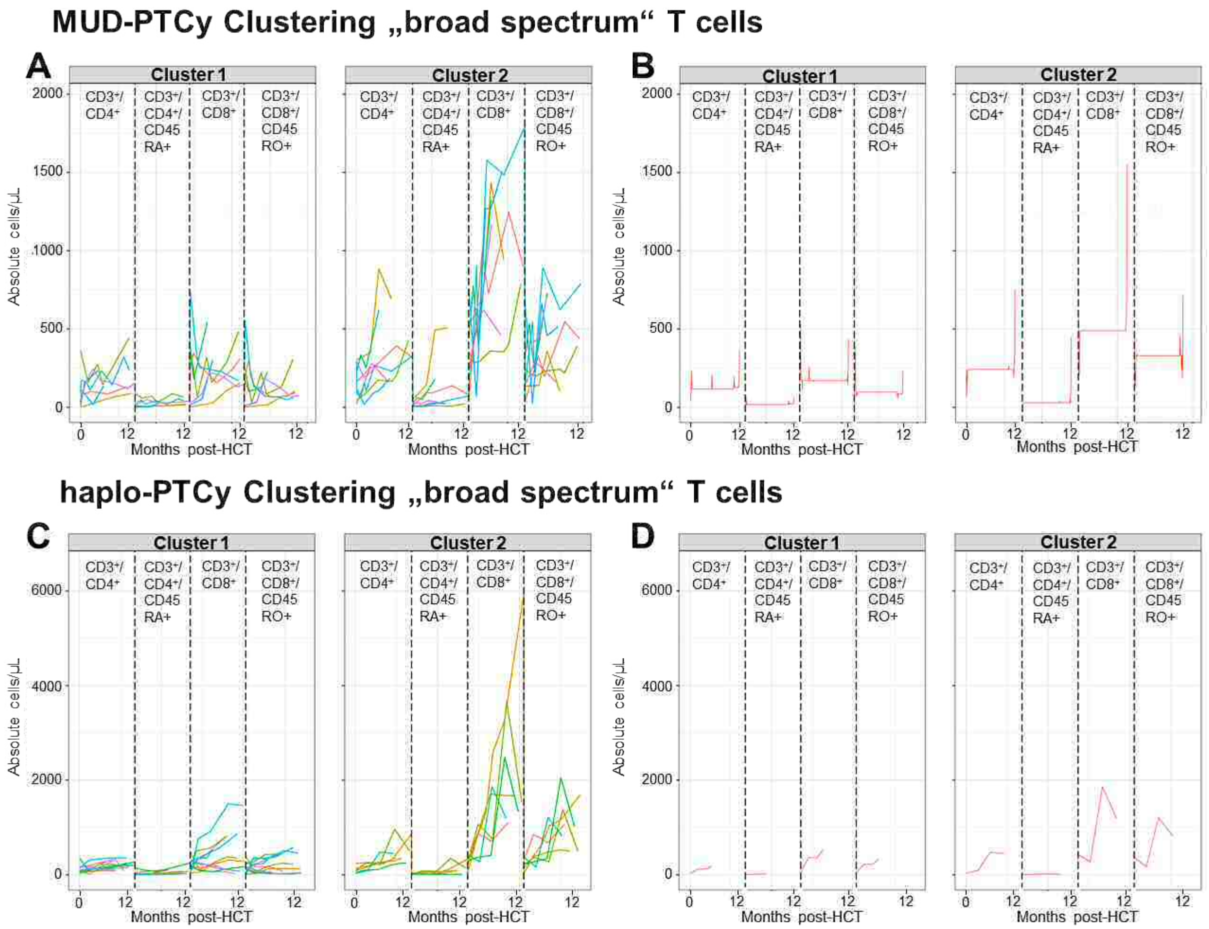

Supplementary Figure 5. Clustering results of PTCy patients in the "broad spectrum" T cell model. (A-D) Individual patient immune cell data clustering in the **(A,B)** MUD-PTCy cohort and **(C,D)** haplo-PTCy cohort using data of "broad spectrum" T cells: $CD3^+/CD4^+$ helper T cells, $CD3^+/CD4^+/CD45RA^+$ naïve helper T cells, $CD3^+/CD8^+$ cytotoxic T cells and $CD3^+/CD8^+/CD45RO^+$ memory cytotoxic T cells, illustrated in distinct boxes. The graphs in **(A,C)** depict each patients' individual reconstitution pattern in the respective subset; **(B,D)** include the medoid samples of each subset calculated via DTW barycenter averaging and partition around medoids, respectively.

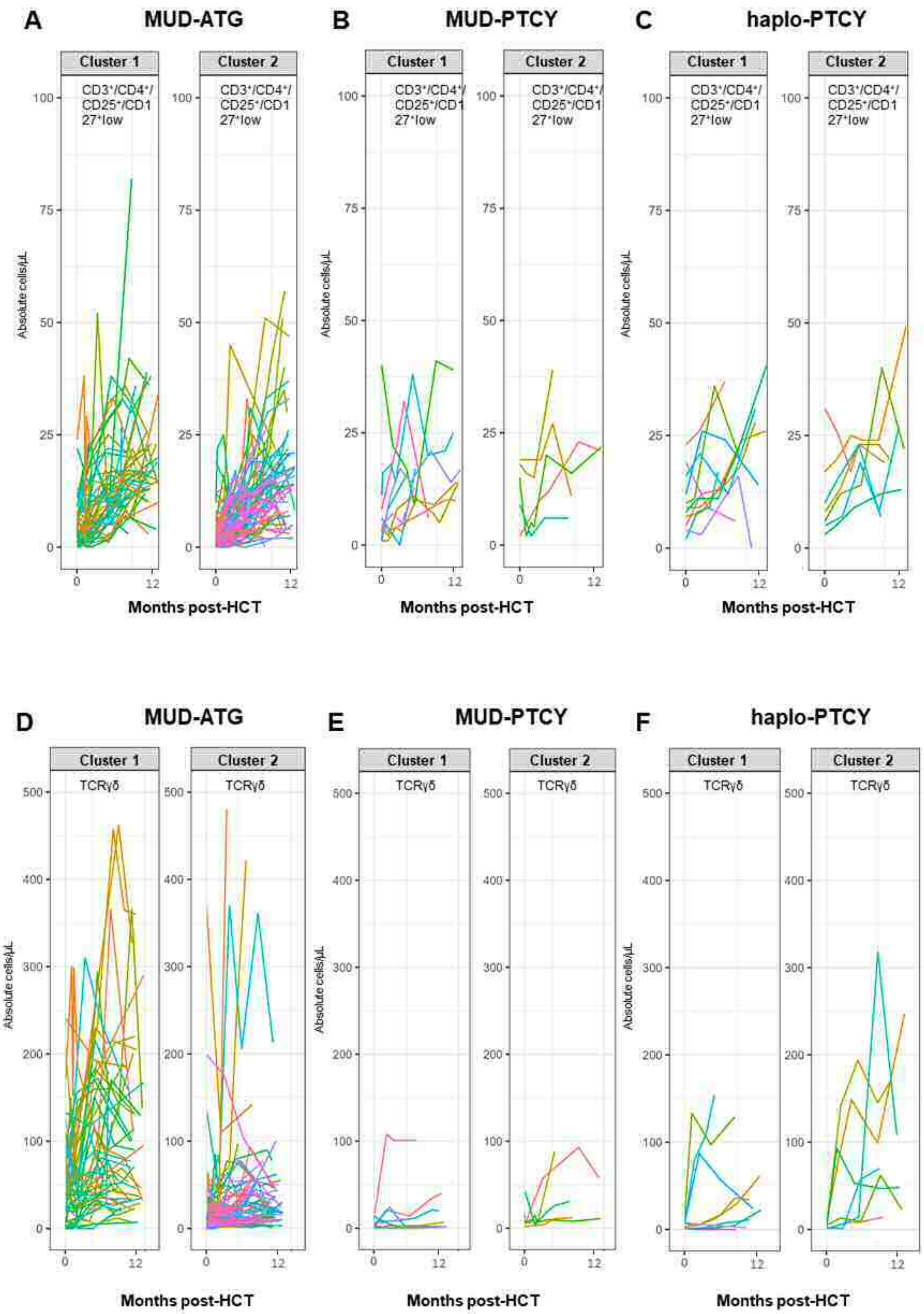

Supplementary Figure 6. Comparative illustration of clustering results in the "GVHD-associated" T cell model. Refined scale results for Tregs and γδ-T cells, which contribute less to the

time-series clustering model than the activated- and αβ-T cells. **(A-C)** Adapted scale illustration of regulatory T-cells (Tregs) in the respective cluster 1 or 2, scale from zero to 100 cells/µl **(A)** MUD-ATG Tregs enlarged from main Figure 4 **(B)** MUD-PTCy and **(C)** haplo-PTCy Tregs enlarged from main Figure 5. **(D-F)** Enlarged illustration of γδ-T cells in the respective cluster 1 or 2, scale from zero to 500 cells/µl. **(D)** MUD-ATG γδ-T cells enlarged from main Figure 4 **(B)** MUD-PTCy and **(C)** haplo-PTCy γδ-T cells enlarged from main Figure 5.

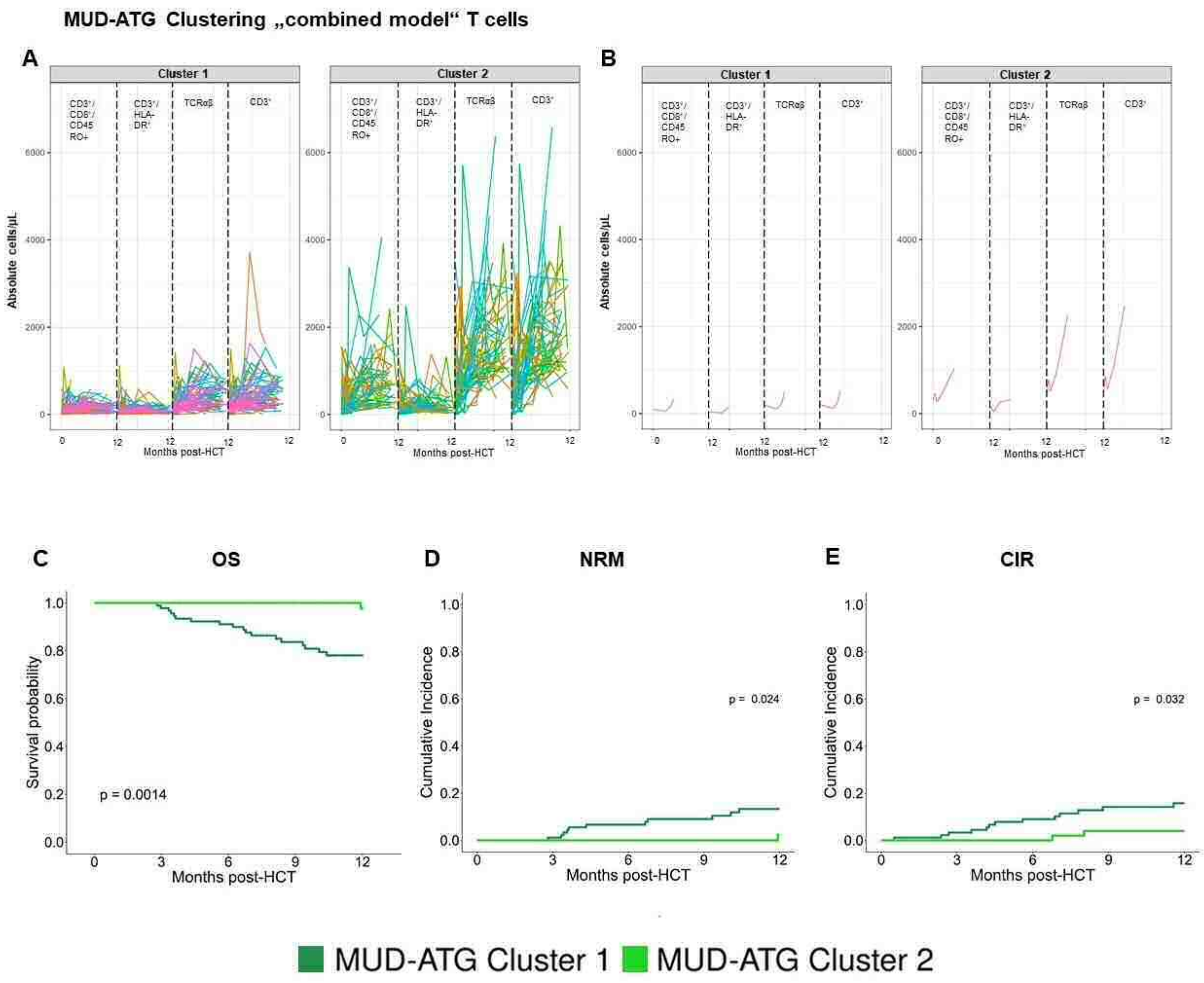

Supplementary Figure 7. “Combined” model using T cell subsets from both the “GVHD-associated” and the “Broad-spectrum” models.

Individual patient immune cell data clustering in the MUD-ATG cohort using a “combined model” with subsets from both “GVHD-associated” and “Broad-spectrum” T cells: CD3+/CD8+/CD45RO+ memory cytotoxic T cells, CD3+/HLA-DR+ activated T cells, TCRα/β+ and CD3+ T cells, The graph in (A) depicts each patients’ individual reconstitution pattern; (B) shows the most representative samples of each T cell subset calculated via partition around medoids (PAM). (C-E) Clinical outcome analysis for the MUD-ATG cohort using the cluster affiliation produced via time-series clustering. (C) Comparison of OS; cumulative incidences of (D) NRM and (E) relapse within 12 months post-HCT.

MUD-ATG - Non-linear spline interpolation preprocessing prior clustering „GVHD-associated“ T cells

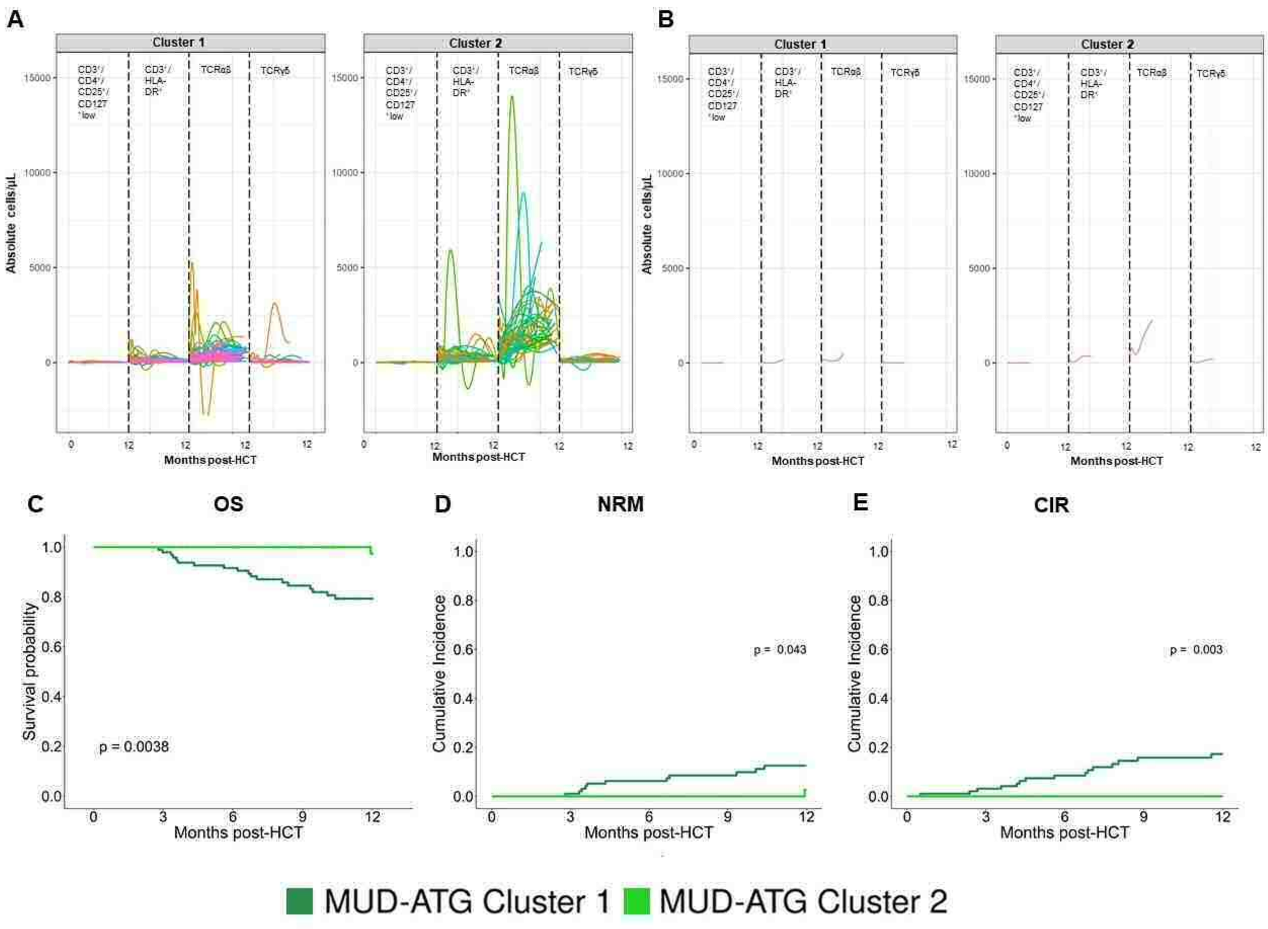

MUD-PTCy - Non-linear spline interpolation preprocessing prior clustering „GVHD-associated“ T cells

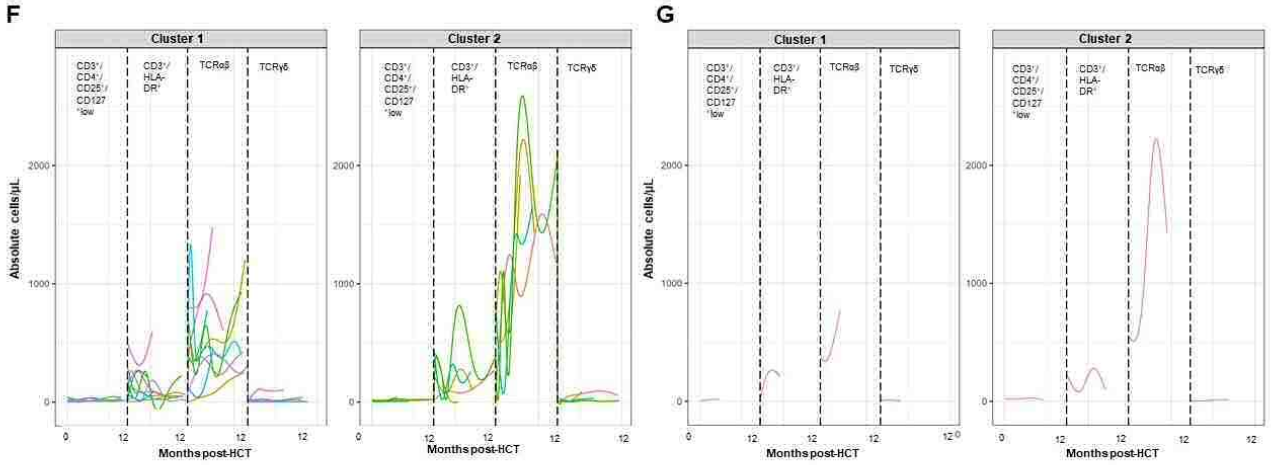

Supplementary Figure 8. Non-linear interpolation in the “GVHD-associated” model

Individual patient immune cell data preprocessed with non-linear interpolation spline interpolation. Clustering in the MUD-ATG cohort using data of “GVHD-associated” T cells:

CD3+/CD4+/CD25+/CD127low regulatory T cells, CD3+/HLA-DR+ activated T cells, TCRα/β+ and TCR γ/δ+ T cells, The graph in (A) depicts each patients' individual reconstitution pattern; (B) shows the most representative samples of each T cell subset calculated via partition around medoids (PAM). (C-E) Clinical outcome analysis for the MUD-ATG cohort using the cluster affiliation produced via time-series clustering. (C) Comparison of OS; cumulative incidences of (D) NRM and (E) relapse within 12 months post-HCT. F-G shows the results for MUD-PTCy patients' individual patient immune cell data preprocessed with non-linear interpolation spline interpolation using the"GVHD-associated" T cells.
